# Future habitat dynamics of critically endangered endemic plants in the St. Catherine protected area, South Sinai, Egypt: climate change perspectives on mountain ecosystems

**DOI:** 10.1186/s12862-025-02408-5

**Published:** 2025-07-10

**Authors:** K Omar, A Mohamed, M. Shaltout, I. Elgamal, L. M Bidak

**Affiliations:** 1 Nature Conservation Secor, Egyptian Ministry of Environment, Cairo, Egypt; 2 United States Naval Medical Research Unit EURAFCENT, Cairo, Egypt; 3 Faculty of Science, University of Alexandria, Alexandria, Egypt; 4 Tilad Environmental Consultation and Veterinary Medicine, Riyadh, Kingdom of Saudi Arabia

**Keywords:** Restricted range species, Conservation monitoring, Prioritization, IUCN red list, Species distribution modeling, Habitat suitability, Spatial changes, Maxent

## Abstract

**Background:**

Mountain ecosystems provide crucial insights into species distribution, yet their fragility, especially in the warming Mediterranean, puts many species at high extinction risk. This study, focusing on four critically endangered plants in Egypt’s St. Catherine Protected Area (*Primula boveana*,* Rosa arabica*,* Micromeria serbaliana*, and *Silene oreosinaica*), uses Species Distribution Models (MaxEnt) and the IUCN Red List to assess climate change impacts and enhance future conservation strategies.

**Results:**

Field observations from 2024 to 2025 revealed changes in Extent of Occurrence (EOO) and Area of Occupancy (AOO) when compared to historical records. EOO increased for all species: *P. boveana* (72.8 km², + 280%), *R. arabica* (102 km², + 117%), *M. serbaliana* (88.5 km², + 30%), and *S. oreosinaica* (61 km², + 15%) as discovery of new and rehabilitated sites. This reclassified *R. arabica* from Critically Endangered (CR) to Endangered (EN), although the other species remain CR. Despite these geographical increases, both human and natural threats continue to cause declines in individual numbers and habitat quality. High predictive model accuracy was recorded (AUC ≥ 0.97, TSS ≥ 0.85). Under current conditions, *P. boveana* and *R. arabica* exhibit wider potential distributions (11.3% and 12.1% of the total area, respectively) than *M. serbaliana* (5.2%) and *S. oreosinaica* (5.4%). Areas with high probability of occurrence are primarily found in the northwestern mountains, often fragmented by topography. MaxEnt projected a decline in suitable habitats for these species, with new suitable areas emerging in SCPA’s southern mountains. Future habitat reduction rates for the years 2050 and 2070 varied: *S. oreosinaica* (2–23%), *P. boveana* (7–32%), and *M. serbaliana* (2–41%), while *R. arabica* demonstrated high stability (> 96%).

**Conclusions:**

Our findings show an altitudinal shift, with species moving to higher, southern mountains, experiencing habitat fragmentation and losses elsewhere. Effective conservation needs ongoing monitoring, in-situ/ex-situ efforts, and addressing threats like overgrazing. Raising environmental awareness is crucial.

**Supplementary Information:**

The online version contains supplementary material available at 10.1186/s12862-025-02408-5.

## Introduction

The Earth system is undergoing significant changes, and human-induced effects on biodiversity have reached geographical significance [1, 2]. The increasingly recognized impacts of these biodiversity changes affect both ecological services and human welfare. Climate change poses a significant threat to biodiversity [3] and has become the primary driver of biodiversity loss, now considered the most pressing global hazard to ecosystems, even surpassing habitat degradation [4]. While ecosystems can typically withstand a certain level of climate unpredictability, rapid alterations are detrimental to biological diversity.

Climate change is anticipated to exacerbate future biodiversity loss [5], making the comprehension of its impacts on biodiversity a critical scientific challenge [4]. Addressing this challenge is important for several reasons. A species’ response to the effects of climate change is determined by its degree of exposure to critical climate variables and its intrinsic vulnerability to these changes [6]. Interactions with other species are also significant [7], as are the cumulative impacts of various external threats within a region [8]. Faced with future climate change, plants may experience three potential outcomes: relocation to newly suitable habitats, adaptation to changing climatic conditions, or an increased risk of extinction [9].

Species range shifts clearly demonstrate an adaptive response of organisms to climate change [10]. In mountainous regions, the total area available at specific altitudes typically decreases with rising elevation. This leads to the upward migration of species along elevational temperature gradients, contributing to the contraction of ranges for numerous species [11, 12]. Climate change could result in extinction events when suitable habitats are unavailable at higher altitudes [13, 14]. Species with distributions restricted to mountaintops are likely among the most vulnerable to habitat loss and subsequent extinction [15, 16]. Recent studies emphasize the importance of predicting range changes within the context of climate change [17, 18] to assess extinction risk [19] and to develop strategies for climate change adaptation and mitigation [20].

High-mountain ecosystems are significant biodiversity hotspots, particularly for endemic species [21–27]. In arid mountainous regions such as Sinai in Egypt, ineffective management and insufficient data have led to detrimental human activities. These include overgrazing, urban expansion, quarrying, and excessive collection of plants for fuel and medicinal purposes, which in turn have caused deterioration in habitat quality, reduction in geographical distribution, and decline in population size [28, 29]. The Egyptian flora includes 49 endemic taxa across 20 families and 45 genera. Notably, the St. Catherine Protected Area (SCPA) in South Sinai is recognized for its significant biodiversity, harboring 16 endemic plant species, which constitute over 32.6% of Egypt’s total endemic flora [30]. Both natural and anthropogenic factors have markedly altered the distribution and habitat quality of these species in this region [22–27, 29, 31, 32]. These studies indicate that cumulative risks will accelerate the extinction of many species with limited geographical distribution. Consequently, the conservation of species in mountainous areas impacted by global warming necessitates careful evaluation and attention, as it relies on the preservation of endemic plants within protected mountainous regions.

*Rosa arabica* Crep., *Primula boveana* Decne ex Duby, *Silene oreosinaica* Chowdhuri, and *Micromeria serbaliana* Danin & Hedge are endemic to the high-altitude areas of SCPA. These species are classified as Critically Endangered (CR) owing to their limited geographical distribution and tiny population size. Furthermore, there is an ongoing deterioration in the quality of their habitats, which is reflected in the observed reductions in both subpopulation numbers and the count of mature individuals [22–25]. Although the conservation status of these species has been studied and published using the IUCN Red List criteria, with identified as Critically Endangered, it is strongly recommended that this status be monitored annually. This monitoring would assess changes in their geographical distribution, population status, and habitats, allowing for the development of action plans adapted to these changes based on the availability of recent field survey information [22–25].

Numerous studies have been conducted to address these challenges, evaluating the potential impacts of climate change on rare and endangered species through various approaches, including field research on global warming, laboratory experiments, and assessments of changes in geospatial distribution [33, 34]. However, a comprehensive understanding of rare and endangered plants’ responses to climate change remains lacking. Consequently, research into the anticipated responses of these species to climate change is becoming increasingly crucial (e.g., [4, 35–37]). Future prediction plays a vital role in informing researchers and policymakers about potential future threats, linking biological changes to climate change, and facilitating the creation of proactive strategies to adapt to and mitigate the impact of climate change on biodiversity [4, 38]. Evaluating the impacts of climate change on habitat distribution at the landscape level is crucial for recognizing potential threats to species range and distribution [39]. The prioritization of conservation projects and the application of successful conservation techniques depend on access to high-quality distribution data [40].

In this regard, the development of effective conservation programs heavily relies on geospatial assessment tools like the IUCN Red List and Species Distribution Models (SDMs) [22]. The evaluation of worldwide biodiversity conservation status largely depends on the IUCN Red List, as its categories and criteria offer a complete and consistent framework for the global assessment of species extinction vulnerability [41]. Data from the Red List encompass many aspects, including geographical distribution, population characteristics, habitat preferences, threats, and suggested conservation strategies. This information helps identify and develop conservation initiatives and recovery plans for species requiring focused efforts [42–44].

On the other hand, particularly in cases with limited geographic data, Species Distribution Models (SDMs) offer a useful means of forecasting the possible geographic distribution of species [22, 23, 45]. SDMs are acknowledged as fundamental methods in ecological modeling used to infer, forecast, and project species distributions and other aspects of biodiversity across various geographical and temporal scales [22, 46]. They serve as primary and significant instruments for evaluating the impact of climate change on the geographical distribution of endemic plant species and as spatial planning tools for the management of conservation projects and field operations [47–50]. By providing effective methods for evaluating habitat suitability across different habitats, these models assist rangers and decision-makers in planning for species recovery, managing spatial range changes, and addressing habitat modifications [51].

In this research, we employed the IUCN Red List criteria to monitor the existing ecological and conservation status of target species. This monitoring aimed to create an adaptive management plan for both in situ and *ex situ* conservation activities in the future. Subsequently, we developed Species Distribution Models (SDMs) to evaluate the impacts of climate change scenarios on the distribution of the target species. This evaluation considered both current and projected climate conditions based on Shared Socioeconomic Pathways (SSPs) for the years 2050 and 2070 across the SCPA. Our analysis included an examination of potential changes in species ranges. The combination of the IUCN Red List criteria and SDMs is deemed crucial for assessing the implications of climate change on species distribution. This approach offers policymakers detailed insights into the present and future conditions of suitable habitats for these species, as well as the anticipated changes (including gains, losses, and stability). Such information is essential for the effective planning and implementation of conservation programs aimed at supporting areas identified as stable or vulnerable.

## Materials and methods

### Study area

**Fig. 1:**
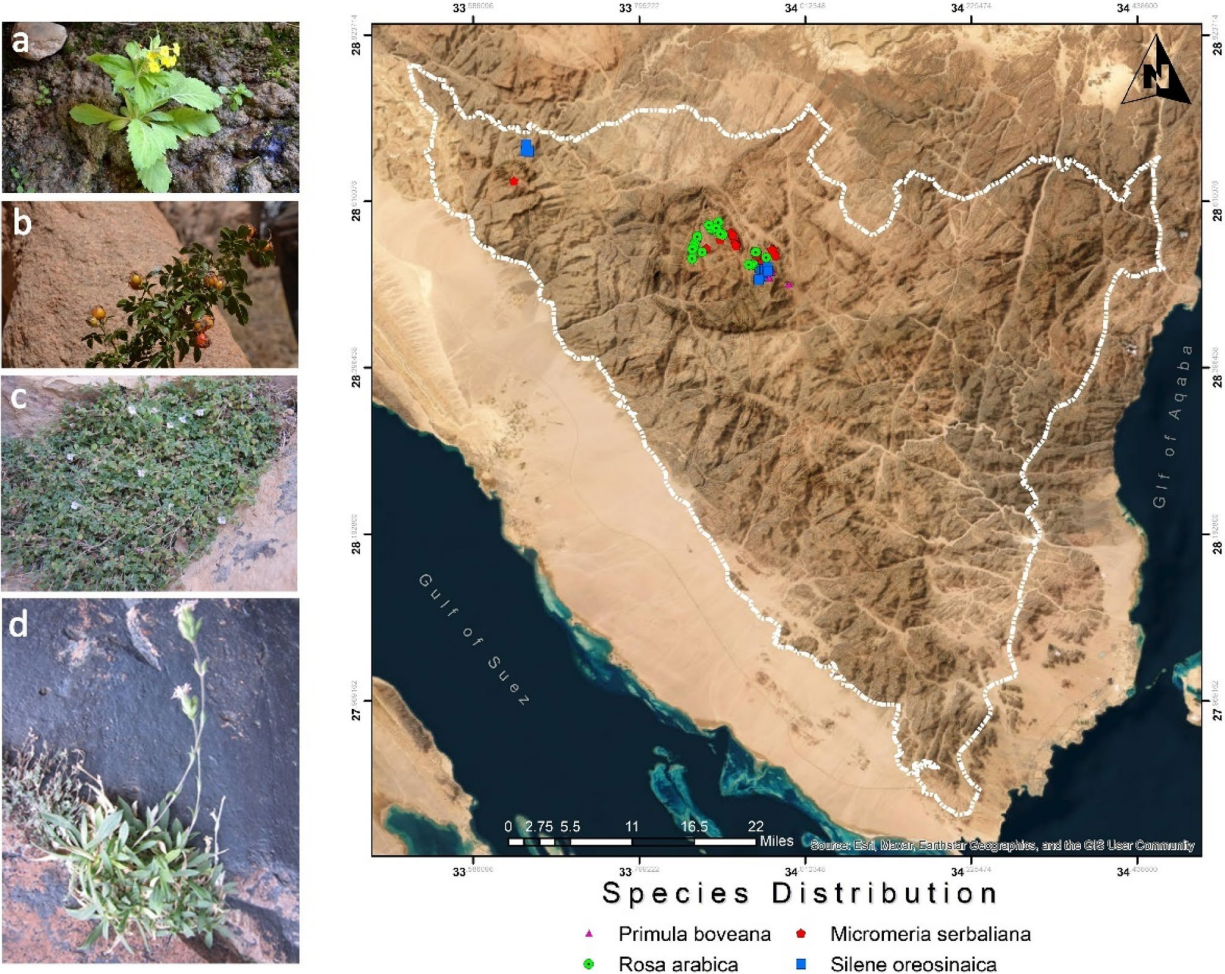
Target species historical distribution within St. Catherine protected area: **a** *Primula boveana* Decne ex Duby; **b** *Rosa arabica* Crép; **c** *Micromeria serbaliana* Danin & Hedge; **d** *Silene oreosinaica* Chowdhuri

Our study area, the St. Catherine Protected Area (SCPA), is situated in the northeastern part of Egypt, specifically in the southern Sinai Peninsula, between 33˚ 55’ and 34˚ 30’ East longitude, and 28° 30’ and 28° 35’ North latitude [52] (Fig. 1). This area, encompassing approximately 4350 km², is entirely located within the mountainous massifs of the South Sinai region. Its complete inclusion within mountain ranges of varying elevation and topography, including Egypt’s highest peak, Mount Catherine (2642 m above sea level), makes it one of the most impressive and resource-rich areas in the Middle East [53]. This topographic heterogeneity supports a unique set of high-altitude ecosystems, harboring a surprisingly diverse fauna and flora with a relatively high representation of endemic species [30, 54]. The flora of these mountains differs from other areas of Sinai due to its unique geology, topography, and climate [55]. The St. Catherine Protected Area stands out as one of the most botanically diverse areas in the Middle East, containing approximately 22% of the Egyptian flora (472 plant species) [56, 57] and over 32.6% (16 endemic species) of Egypt’s endemic plant species [30].

Considered a major hotspot for plant diversity in the Middle East within the Saharo-Sindian (Irano-Turanian) region, the South Sinai Mountains are characterized by a Sahara-Mediterranean climate in their southern desert area [58]. While winters are rather cold, with an average minimum temperature of −7.8°C in February, summers are rather hot, with an average maximum temperature of 36°C in August, although altitude moderates the temperature regime [23]. Apart from sporadic winter snow, orographic factors can increase precipitation to 300 mm per year at higher altitudes [54, 59]. Recognized as an Important Plant Area (IPA), an Important Bird Area (IBA), and most recently a Key Biodiversity Area (KBA), these special environmental conditions have led to the isolation of some endemic plant species and the development of notable spatial concentration and fragmentation in the high mountain zone (500 to 2600 m), which will be the focus of our study [30].

### Target species

A total of four endemic plant species were selected for this study within the St. Catherine Protected Area (SCPA). The selection was based on their IUCN Red List conservation status, critical distribution range, ecological importance, availability of historical data, accessibility, and the degree of threats they face. These species are *Primula boveana* Decne ex Duby, *Micromeria serbaliana* Danin & Hedge, *Rosa arabica* Crép., and *Silene oreosinaica* Chowdhuri. Detailed notes about each of these species follow:

- *Primula boveana* Decne ex Duby
  *Primula boveana* Decne ex Duby, belonging to the family Primulaceae and commonly known as the Sinai primrose, is notable for its unique reproductive biology and significant rarity (Fig. 1a). This endemic species is confined to the Saint Catherine Protectorate Area (SCPA) in the mountainous region of southern Sinai, Egypt, where it is found at elevations exceeding 1,700 m [60]. It is primarily restricted to five specific locations surrounding Mount Catherine. The population size is notably limited, comprising fewer than 200 individuals. *Primula boveana* is characterized as “one of the rarest plant species” due to its highly restricted distribution, rarity, and ongoing rapid decline [61]. This species is listed as Critically Endangered on the IUCN Red List due to its highly restricted range, with an extent of occurrence (EOO) of 9.8 km² and an area of occupancy (AOO) of under 16 km² [60]. It is restricted to montane wadis supported by melting snow and is found in moist soils adjacent to wells and springs [60].
  Documented evidence indicates a persistent decline in habitat quality for this species, accompanied by reductions in subpopulation sizes and the number of mature individuals, as well as significant fragmentation [23, 59, 60]. Additionally, climate change is anticipated to intensify habitat loss for this high-elevation species [60]. It is classified as a national conservation priority species in Egypt due to its extreme rarity, highlighting the urgent need for the implementation of both in situ and *ex situ* conservation measures [62, 63].
- *Rosa arabica* Crép.
  *Rosa arabica* Crép. is a perennial shrub in the Rosaceae family (Fig. 1b). This species is endemic to the high mountain region of the SCPA in southern Sinai, Egypt, and is found within a specific elevational range of 1,700 to 2,350 m a.s.l [31, 64–66]. The branches may extend to 3 m in length, featuring uniform prickles and obovate leaflets that are deeply double-serrate, sparsely glandular on the upper surface, and hairless. Flowering occurs in late spring, while seed reproduction occurs in late summer [66–68]. Despite its small population size (less than 100 individuals), it is collected for several local uses, such as in traditional medicine where its flowers and leaves act as a pain reliever for menstrual pain [66, 69]. It is also harvested for firewood and garden fence construction and has economic importance as a pastoral resource for camels and donkeys [66].
  The species’ limited geographical distribution, coupled with overexploitation, has led it to the verge of extinction [66]. This species is limited to one main location and is especially found among 13 subpopulations covering less than 40 km², with a fragmented population and challenges for natural seed germination [66, 69]. The main threats to this species are identified as long-term drought, overgrazing, overcollection, climate change, and sudden floods. Consequently, *Rosa arabica* is listed as Critically Endangered [66].
- *Micromeria serbaliana* Danin & Hedge
  *Micromeria serbaliana* (syn. *Satureja serbaliana* (Danin & Hedge) Greuter & Burdet) is a narrowly endemic and threatened species belonging to the family Lamiaceae ([62]– Fig. 1c). It is endemic to the SCPA in South Sinai, Egypt. According to historical sources, its distribution is limited entirely to high mountain regions above 1700 m. This plant has a limited geographic range of less than 40 km² and occupies microhabitats on rocky cliffs and slopes. It has been observed that this species has experienced notable fragmentation and fluctuations during the previous 50 years [26]. Originally identified by Avinoam Danin in 1968, it was rediscovered in 1998 [70] and most recently observed in 2017 [26]. Overgrazing exposes this species to the loss of fruiting and floral parts, which has resulted in a decline in its numbers and the degradation of its habitat, similar to other endemic species in the same area [31, 71–74]. The species is classified as Critically Endangered [26].
- *Silene oreosinaica* Chowdhuri
  *Silene oreosinaica* Chowdhuri is a plant species endemic to the SCPA, belonging to the family Caryophyllaceae. It is a very rare species [62]. Due to its very limited distribution, with an EOO of less than 25 km² and an AOO of about 8 km², it is restricted to only two sites and confined to rocky slopes and cliffs. Its population is under extreme risk from overgrazing, is highly fragmented, and its habitat quality is declining [27]. In addition, climate change is expected to reduce the habitat available to this high-altitude specialist. For these reasons, this species is classified as Critically Endangered [27].

### Data collection and analysis

#### Data sources and sampling techniques

This study employed various methods to acquire precise and current data on the target plants, thereby reducing gaps in the analysis process. These methods involved the collection and extraction of data from prior studies focused on the same target species [22–27, 29, 54, 56, 57, 59, 60, 66, 70, 75–77], as well as from the fieldwork database of Omar and Elgamal (2014–2021). This database will serve as a baseline for the comparison and monitoring of geographical distribution, population size, and habitat status changes.

Historic sites for the target species were mapped, and potential habitats were identified utilizing ArcGIS 10.9. Research on the distribution of target endemic plants within the SCPA indicates that all such plants are situated within the high mountain area, specifically in the northern central part of the SCPA [22–27]. Between May 2024 and April 2025, a total of 30 primary sites in high mountain areas were surveyed for verification. The objective of the field survey was to provide an accurate description and updates on the distribution, geographical extent, and area of occupancy of the target species in relation to their environmental requirements, rather than to conduct a broad vegetation study of the area. This survey is carried out every two years. This information is essential for determining the Red List Status [41]. A systematic sampling approach was employed to capture local environmental gradients by placing 50 quadrats, each with an area of 50 m², in selected areas using quadrat sampling techniques [78]. The selection of quadrat locations was informed by historical data, the presence of target species identified during the field survey, and the discovery of new areas based on suggestions from previous work [22–25]. The altitudinal range for this survey spanned from 500 to 2400 m a.s.l. and encompassed all microhabitats according to historical data. A total of 200 distribution points were collected from all sources, with 85% of them recorded during fieldwork. These points then underwent a cleaning process to remove duplications. This resulted in a final set of 126 unique distribution points. These points belonged to *Rosa arabica* Crep. (33 points), *Primula boveana* Decne ex Duby (25 points), *Silene oreosinaica* Chowdhuri (30 points), and *Micromeria serbaliana* Danin & Hedge (38 points).

Based on the preceding steps, the ecological and conservation status has been thoroughly assessed in accordance with IUCN Red List standards [22–27]. Adhering to the IUCN Red List Assessment requirements, data on geographical distribution, population status, habitat and ecological characteristics, threats, and conservation requirements for the target species were collected, extracted, and updated.

### IUCN red list assessment

In this step, we employed the IUCN Red List Categories and Criteria: Version 16 [41] and guidelines [79] to assess the current ecological and conservation status of these species. This approach serves as a global standard tool for collecting the following data:

- Geographic range: During the field survey within the SCPA, we documented the target species. A GPS fix was obtained for 50 quadrats in decimal degrees using the World Geodetic System 1984, with the correction documented to the fifth decimal place. The altitudinal range was measured in meters above sea level. ArcGIS 10.9 was utilized to plot the study sites. In accordance with IUCN Standards and Petitions Committee [79], we documented the number of locations where the target species was found, along with the Extent of Occurrence (EOO) and Area of Occupancy (AOO) using a 2 × 2 km grid. The Geospatial Conservation Assessment Tool (GeoCAT - http://geocat.kew.org/) was utilized to compute and visualize the EOO and AOO.
- Population characteristics: In each quadrat, the total number of individuals and the number of mature individuals were recorded to calculate the size of the subpopulations and the overall population of the target species. The data regarding local population sizes serves as a foundational reference for subsequent comparisons aimed at documenting trends, fluctuations, and fragmentation within populations. The estimation of population size and the number of mature individuals was calculated based on the accuracy of data and the levels of uncertainty outlined in the IUCN Standards and Petitions Committee [79].
- Habitats and ecology: In each quadrat, the preferred ecological setting of the target species was documented in accordance with the IUCN Habitats Classification Scheme version 3.1. Microhabitat types and status within the study area were documented, including slope, wadi, terraces, gorge, and cave, etc. Climatic features, including maximum temperature, minimum temperature, and precipitation, for the average years 2010 to 2024 were obtained from the SCPA weather station. Soil samples were collected from each quadrat at depths of 0–30 cm. In certain mountainous locations where the soil was notably shallow, maximum sample depths ranged from 10 to 20 cm. The analysis of physical and chemical properties, including texture, pH, electrical conductivity (EC µs/cm), total dissolved solids (T.D.S PPM), water content percentage, organic matter percentage, calcium carbonate percentage, sodium (Na + PPM), calcium (Ca + + meq/L), potassium (K + PPM), magnesium (Mg + + meq/L), bicarbonate (HCO3- meq/L), sulfate (SO4– meq/L), and chloride (Cl- meq/L) was conducted [80–82]. The vegetation characteristics of the target species, including density, cover, abundance, and associated species within each quadrate, were recorded [83].
- Threats: During the field survey, we documented all activities potentially contributing to the decline of plant populations, including grazing, collection, drought, water abstraction, and the presence of invasive species [71, 84]. The IUCN Threats Classification Scheme 3.3 was used to assess the timing, scope, severity, and impact score for each threat [22–27].
- Conservation requirements: This section compiles all available data regarding past, ongoing, and future activities aimed at conserving these species through in situ or *ex situ* practices. It also includes recommended conservation actions, requirements, and research necessary for the preservation of the target species, as outlined by the IUCN Standards and Petitions Committee (2019).
- Extinction risk: The degree of vulnerability of the target species to extinction is determined using the IUCN Red List Categories and Criteria Version 16, as outlined in Table 2.1 on Page 16 of the IUCN Standards and Petitions Committee [79]. We utilized criterion B, specifically “B1 (extent of occurrence) and/or B2 (area of occupancy)” to evaluate our field data. Previous status was compared with this study to determine the spatial changes and possibilities of changes in conservation status categories.

### Species Distribution Model (SDM)

To identify and extract suitable habitat for the target species under prevailing conditions, we undertook the following steps:

- *Species coordinates*
  Before starting the analysis, the provided data (previous work and fieldwork) underwent preprocessing. During this stage, duplicate entries and records with incomplete longitude (Y) and latitude (X) were deleted, resulting in a final set of 126 unique distribution recording points. These points belonged to *Rosa arabica* Crep. (33 points), *Primula boveana* Decne ex Duby (25 points), *Silene oreosinaica* Chowdhuri. (30 points), and *Micromeria serbaliana* Danin & Hedge (38 points). To reduce clustering effects in model predictions, and in accordance with standard resolution practices [85, 86], a single data point was retained within each 1 km × 1 km grid cell. All verified distribution data points for the four target species were retained for the final analysis to generate potential distributions using MaxEnt modeling. The occurrence-point data were imported into Microsoft Excel according to MaxEnt model requirements, saved in CSV format, and sorted by species name, longitude, and latitude.

b. *Environmental variables*
  We utilized all available coordinates to develop a predictive model for the potential distribution of the target species. The distribution of plant species is influenced by a variety of environmental factors, including climatic, topographic, edaphic, and anthropogenic elements [22–27]. Consequently, we considered thirty environmental variables as potential predictors of the target species’ habitat distribution. The dataset comprises 19 bioclimatic variables, 3 topographic variables (elevation, aspect, and slope), and 8 soil variables (bulk density, clay content, sand, silt, coarse fragments, water content, organic carbon, and soil pH). The selection of these variables was informed by their biological significance to the distribution of the target species and by other habitat modeling studies conducted in the same region (e.g., [22–27, 29–31, 54–57, 68, 73, 75, 76, 87–92]) (Table 1).

**Table 1:** Environmental variables used in the study

Code	Environmental variables	Unit		Code	Environmental variables	Unit
Bio1	Annual mean temperature	°C		Bio16	Precipitation of wettest quarter	mm
Bio2	Mean diurnal range	°C		Bio17	Precipitation of driest quarter	mm
Bio3	Isothermality (Bio3/Bio7)	°C		Bio18	Precipitation of warmest quarter	mm
Bio4	Temperature seasonality	\		Bio19	Precipitation of coldest quarter	mm
Bio5	Max temperature of warmest month	°C		Elevation	Elevation	m
Bio6	Min temperature of coldest month	°C		Aspect	Aspect	◦
Bio7	Temperature annual range	°C		Slope	Slope	◦
Bio8	Mean temperature of wettest quarter	°C		pH	Soil pH	ph*10
Bio9	Mean temperature of driest quarter	°C		O.C.	Soil organic carbon	dg.kg
Bio10	Mean temperature of warmest quarter	°C		Clay	Soil clay content	g/kg
Bio11	Mean temperature of coldest quarter	°C		Bulk	Soil bulk density (kg/dm3)	cg/cm3
Bio12	Annual precipitation	mm		Sand	Sand	cg/cm3
Bio13	Precipitation of wettest month	mm		Silt	Silt	cg/cm3
Bio14	Precipitation of driest month	mm		Coarse	Soil Coarse fragments	cg/cm3
Bio15	Precipitation seasonality	mm		W.C.	Soil Water Content	mm/m
  Nineteen bioclimatic variables (Bio1–Bio19), which are biologically significant for defining the eco-physiological tolerances of a species [22, 93], were obtained from the WorldClim database for current climatic conditions. These data feature a spatial resolution of 30 arc-seconds (approximately 1 km²). Environmental factors for three periods—the current period (1970–2000), 2040–2060 (2050), and 2060–2080 (2070)—were obtained from WorldClim (http://www.worldclim.org/), which includes both current data and projections for future periods (2021–2100). The socioeconomic scenario pathways SSP12.6 (sustainability) and SSP58.5 (fossil fuel development) from the IPSL-CM6A-LR global general circulation model (GCM) were used as future climate projections.
  The global climate model IPSL-CM6A-LR, developed at the Institut Pierre-Simon Laplace (IPSL) to study natural climate variability and climate response to natural and anthropogenic forcings, is part of the sixth phase of the Coupled Model Intercomparison Project (CMIP6). The recent IPSL-CM6A-LR model climatology shows significant improvement over its previous version (CMIP5) [94]. This study employed the IPSL-CM6A-LR climate model, recognized for its efficiency in global climate projections, to assess the effects of future climate change on the distribution of plant and animal species (e.g., [95–98]). The IPSL-CM6A-LR climate model has been effectively evaluated in desert ecosystems (e.g., [99–103]).
  Soil characteristics, including bulk density, clay content, sand, silt, coarse fragments, water content, organic carbon, and soil pH, for the top 5–15 cm layers were obtained from www.soilgrids.org [104]. Elevation data with a resolution of 1 km² were acquired from the Shuttle Radar Topography Mission (SRTM). This data was utilized to compute slope, aspect, curvature, and area solar radiation, among other metrics, using the Spatial Analyst tool in ArcGIS 10.9 software.
  Geographic coordinates are expressed in decimal degrees (to five decimal places) according to the WGS 84 standard. These coordinates were spatially represented and verified using Google Earth. The target area layer was extracted after downloading files spanning 1950–2000, utilizing an SCPA boundary mask. The file was subsequently converted to ASCII format using the latest version of ArcGIS for compatibility with MaxEnt. Autocorrelation analysis was performed on the spatial data to eliminate spatially correlated data points, addressing the record linkage issue [105, 106]. Duplicate records were eliminated, retaining a single record within a grid encompassing an area of approximately 1 km² [107].
  Multi-collinearity in regression analysis: A set of identified environmental variables was tested for collinearity. Correlation analysis on all environmental variables (i.e., climate variables, topographic features, and soil properties) was performed in R version 4.2.1 using the ‘caret’ package. During this analysis, if Pearson’s correlation coefficient between any pair of variables was greater than 0.75, one variable from that pair was selected for exclusion [108, 109]. Environmental variables were further reduced through trial runs in a maximum entropy method (Maxent) using permutation importance and jackknife tests (Table 2). Research indicates that excessive specification of parameters, along with a decrease in predictive power and interpretability, may arise from the inclusion of numerous interrelated variables [110–112].

**Table 2:** Contributions, importance gains of the environmental variables used in MaxEnt modelling of target species in the study area

	Primula boveana		Rosa arabica		Micromeria serbaliana		Silene oreosinaica	
Code	**P.C (%)**	**P.I (%)**	**P.C (%)**	**P.I (%)**	**P.C (%)**	**P.I (%)**	**P.C (%)**	**P.I (%)**
Bio2			4.6	10.2				
Bio3					8.6	2.1		
Bio4	3.1	21.7						
Bio6			1	31.3				
Bio8	30	59.3			55.3	90.5	31.3	86.2
Bio11	7.5	16.9						
Bio12	47.9	0	0.2	0.2	26.6	0	2.9	0
Bio13							55.3	0
Bio19			84.5	44.6				
Elevation	8.3	0	0.8	1	0.7	0	1.7	0
Aspect					5.3	1.6		
Slope	1.7	0.6						
O.C.			1.1	2.2				
Clay	1.5	1.5			1.8	1.5		
Bulk							2.4	5.6
Coarse			1.1	1.2	1.7	4.3	6.2	7.3
W.C.			6.6	9.3			0.2	0.9

Percent Contribution (P.C), Premutation Importance (P.I)

c. *Modeling procedure*
  The maximum entropy distribution (MaxEnt) modeling technique demonstrates superior performance compared to various other methods [23, 45, 84, 113] and remains effective even with limited sample sizes [22, 114, 115]. However, ensemble modeling has been gaining momentum in species distribution modeling (SDM) over the past decade. This approach involves combining predictions from single SDM models into one predicted binary map, which many studies suggest may be more accurate. Nevertheless, single-algorithm modeling methods, particularly MaxEnt, are capable of producing distribution maps with comparable accuracy to ensemble methods. Furthermore, the ease of use, reduced computational time, and simplicity of methods like MaxEnt support their use in scenarios where the choice of modeling methods, knowledge, or computational power is limited, but the need for robust and accurate conservation predictions is urgent [116, 117]. This study necessitates only species presence data and environmental variable layers, which can be continuous or categorical, for the designated area. MaxEnt software, version 3.4.4, was utilized to estimate the probability of species presence, ranging from 0 (unsuitable) to 0.99 (optimal habitat suitability). The current study utilizes ASCII files for the selected environmental variables and a CSV file for species presence localities in decimal degrees. For each species, models ranging from 5 to 15 were tested, with the model most representative of the current situation selected for comparison with future climatic conditions. A total of 80% of the location point data was used for training, and the remaining 20% to test the predictive ability of the model. Ten replicates were considered. All other parameters remained in their default settings, which relates to the probability of suitable conditions, ranging from 0 to 1. Average and standard deviation values for training and test AUC for the 10 models were extracted from the Maxent text result output.
d. *Model validation*
  The evaluation of MaxEnt’s performance was conducted using a threshold-independent Receiver Operating Characteristic (ROC) analysis and Area Under the Receiver Operating Characteristic Curve (AUC) values, which range from 0.5 (indicating random discrimination) to 1 (indicating perfect discrimination) [118, 119]. True Skill Statistic (TSS) was also utilized; it is calculated as sensitivity plus specificity minus one, with values ranging from − 1 to 1. Values exceeding zero signify superior model performance relative to chance [120]. From the MaxEnt results (maxentResults.csv), the maximum training sensitivity plus specificity (MTSS) was used to select the threshold selection method for evaluating binary classification models. This method aims to find a probability threshold that optimally balances the model’s ability to correctly predict the presence of a species (sensitivity) and its ability to correctly predict the absence of a species (specificity) based on the training data. It combines sensitivity (the proportion of correctly predicted presences) and specificity (the proportion of correctly predicted absences): *TSS = Sensitivity + Specificity* − 1.
  R version 4.2.1 was employed to calculate the True Skill Statistic (TSS) values, which ultimately span a range from − 1 to + 1 [120]. A TSS of + 1 indicates perfect agreement between the model’s predictions and the observed species occurrences. A value of 0 suggests that the model performs no better than random chance. Conversely, a TSS of −1 implies that the model’s predictions are completely opposite to the actual observed pattern of species distribution. Mean AUC and TSS across the 10 replicates of each algorithm and across all species were used to assess model performance. A total of 1000 iterations were chosen to allow sufficient time for model convergence, with a threshold of 0.00001. We assessed the relative importance of each environmental predictor in the model by utilizing the percentage contribution from the Jackknife test, which is considered the most appropriate index for small sample sizes [121].
e. *Define spatial changes*
  Ten replicates were used, and the average and standard deviation for training AUC, test AUC, and TSS across the ten models were obtained from the MaxEnt text output [22–27]. Subsequently, the average model for the target species was imported into ArcGIS. The selected logistic output format represents the probability of suitable conditions, ranging from 0 to 1. The habitat suitability predictions were categorized into four classes: ≥0.71 (High Probability), 0.31–0.70 (Moderate Probability), 0.11–0.30 (Low Probability), and ≤ 0.10 (Very Low to Unsuitable for species presence) [122]. The area size for each category was then determined using ArcGIS 10.9.
  To further analyze spatial changes resulting from future climate change, we assessed distribution shifts between current suitable habitats and two future scenarios within the ArcGIS environment. Initially, based on the species’ ecological characteristics, the continuous model map was transformed into a binary map (1: suitable/0: unsuitable) by applying a threshold value ranging from 0.3 to 0.5. This threshold was determined based on MTSS, ecological knowledge, and the species’ habitat requirements, and the conversion was performed using ArcGIS 10.9. Instead of relying solely on statistical criteria (such as maximizing a model evaluation metric) or arbitrary cut-offs, this approach incorporates ecological knowledge to make more informed decisions about what constitutes a “suitable” habitat for the species, drawing upon long-term field experience. The change in suitable areas was calculated by finding the difference between current and future climatically suitable areas [22, 23, 123]. The rates of gain and loss were calculated by comparing the respective areas to the total area of the species’ distribution under current climatic conditions (percentage of gain or loss = (gained or lost area/total suitable area under current climate conditions) * 100).

## Results

### Geographic range

**Fig. 2:**
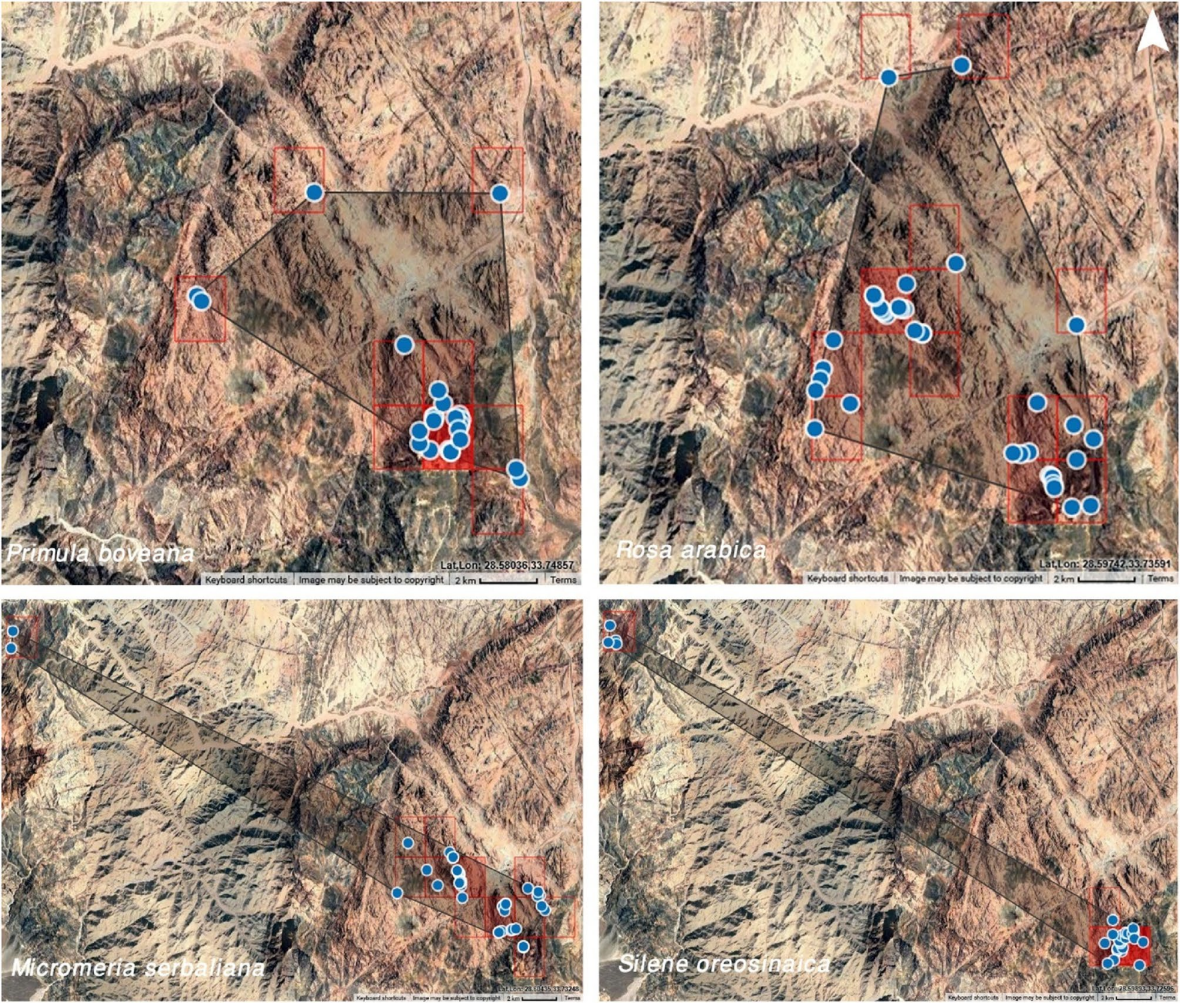
Updated geographical distribution of target species within SCPA (survey 2025)

During the field survey, all target species were documented within a specific elevation range of 800–2400 m above sea level. This survey recorded new sites, not previously documented, in addition to the rehabilitated sites that have been carried out in 2021 particularly for *Primula boveana* and *Rosa arabica*, located north of their historical distribution (Fig. S1). Consequently, both the Extent of Occurrence (EOO) and Area of Occupancy (AOO) increased compared to the pre-study period. The distribution range of *Primula boveana*, *Rosa arabica*, *Micromeria serbaliana*, and *Silene oreosinaica* reached 72.8 km^2^ (18.6 km^2^ before this study), 102 km^2^ (47 km^2^), 88 km^2^ (68 km^2^), and 61 km^2^ (53 km^2^), respectively, while the Area of Occupancy (AOO) reached 36 km^2^ (24 km^2^), 52 km^2^ (36 km^2^), 48 km^2^ (44 km^2^), and 16 km^2^ (12 km^2^), respectively (Table 3). Species distribution and number varied; *P. boveana* and *R. arabica* occurred exclusively in the “high mountain zone.” In contrast, *M. serbaliana* and *S. oreosinaica* were recorded in two different locations: the Serbal area and the high mountain area, extending east to west across South Sinai (Fig. 2).

**Table 3:** Monitoring of geographical distribution characteristics of target species

Scientific name	EOO (km^2^)		AOO (km^2^)		Geographic distribution	Altitude range (m)	No of Populations
	Before	After	Before	After			
*Primula boveana*	18.5	72.8	24	36	High Mountain Area	1700–2350	1
*Rosa arabica*	47	102	46	52	High Mountain Area	1500–2400	1
*Micromeria serbaliana*	68	88	40	48	High Mountain Area– Serbal Area	1700–2200	2
*Silene oreosinaica*	53	61	12	16	High Mountain Area– Serbal Area	900–2300	2

### Population information

**Fig. 3:**
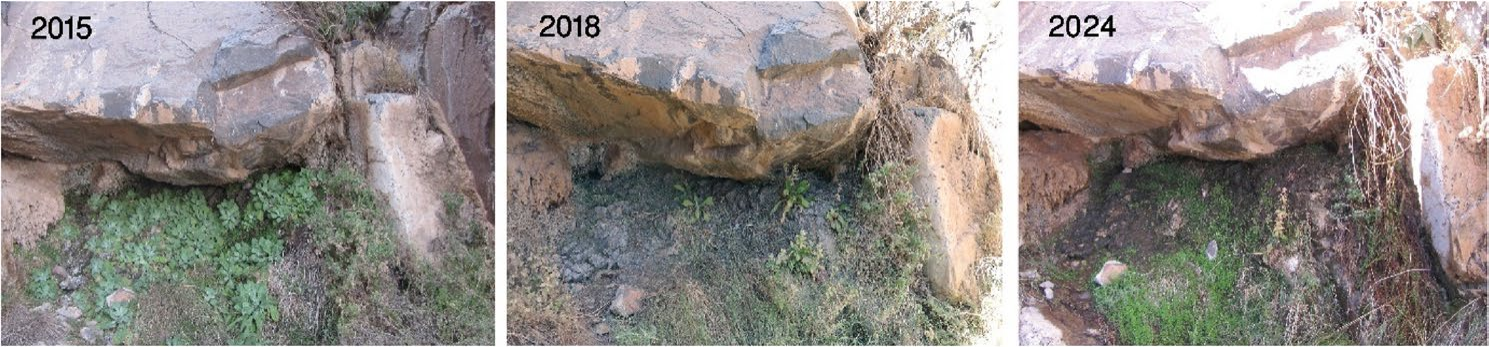
Population size trend and fluctuation in ten years for *P. boveana*

**Fig. 4:**
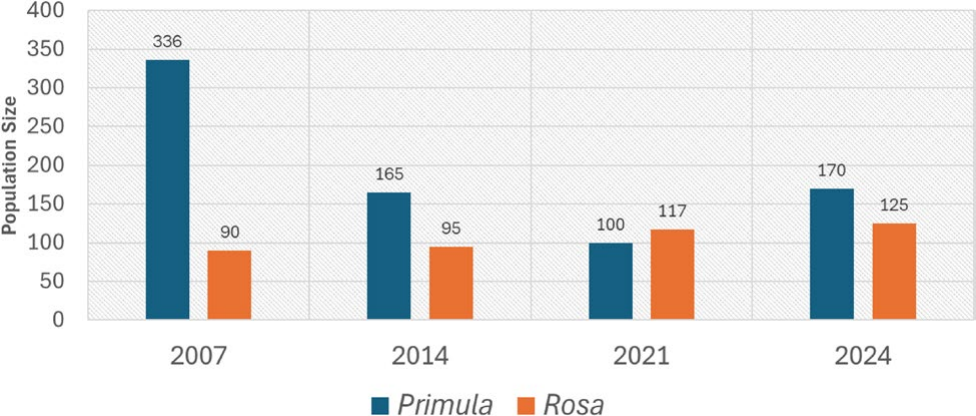
Population size trend and fluctuation for *P. boveana* and *R. arabica*

During both the desk review and current field survey, the targeted species were found in very small, discontinuous, and scattered sub-populations in areas with rocky soil. Individuals were observed singly and far apart, their distribution influenced by the specific micro-habitat. As a result of recording new sites (2 for *Primula* and 3 for *Rosa*), the estimated population size was approximately 170 for *P. boveana*, 125 for *R. arabica*, 270 for *M. serbaliana*, and 130 for *S. oreosinaica*. Although *P. boveana* and *R. arabica* were found in only one area (high mountain area), and *M. serbaliana* and *S. oreosinaica* in two locations, there are clearly separate sub-populations for each species due to mountain barriers (10 for *P. boveana*, 20 for *R. arabica*, 10 for *M. serbaliana*, and 3 for *S. oreosinaica*). The number of mature individuals within these sub-populations varied, ranging from 1 to 32 for *R. arabica*, from 3 to 400 for *P. boveana*, from 1 to 18 for *M. serbaliana*, and from 1 to 8 for *S. oreosinaica* (Table 4). The new sites recorded in this survey were very close to the rehabilitation sites for these target species that occurred in 2020. Based on previous studies, the population trend, fluctuation, and decline rate were calculated, confirming that these sub-populations experience fluctuations and instability (Figs. 3 and 4, and S2).

**Table 4:** Population size and fluctuation trend of each subpopulation of target species (Total and ± SD)

	Rosa		Primula		Micromeria		Silene	
Site No	Frequency	No. Individuals	Frequency	No. Individuals	Frequency	No. Individuals	Frequency	No. Individuals
1	1	2 ± 0	1	7 ± 0	1	3 ± 0	4	1 ± 0
2	7	40 ± 2.8	1	8 ± 0	1	3 ± 0	11	3 ± 0.7
3	3	5 ± 0	1	20 ± 0	1	1 ± 0	30	8 ± 2.4
4	1	2 ± 0	4	45 ± 8.1	1	3 ± 0		
5	3	6 ± 0	8	410 ± 42.8	5	12 ± 3.7		
6	3	10 ± 0.8	1	50 ± 0	9	11 ± 3.2		
7	1	1 ± 0	2	28 ± 16.2	5	18 ± 6.6		
8	1	1 ± 0	1	3 ± 0	5	8 ± 2.6		
9	1	1 ± 0	1	20 ± 0	3	17 ± 7		
10	1	2 ± 0	1	15 ± 0	4	3 ± 0.9		
11	1	6 ± 0.2						
12	3	4 ± 0						
13	2	4 ± 0						
14	1	1 ± 0						
15	6	22 ± 1.5						
16	2	2 ± 0						
17	3	9 ± 0.5						
18	1	1 ± 0						
19	1	1 ± 0						
20	1	1 ± 0						

**Fig. 5:**
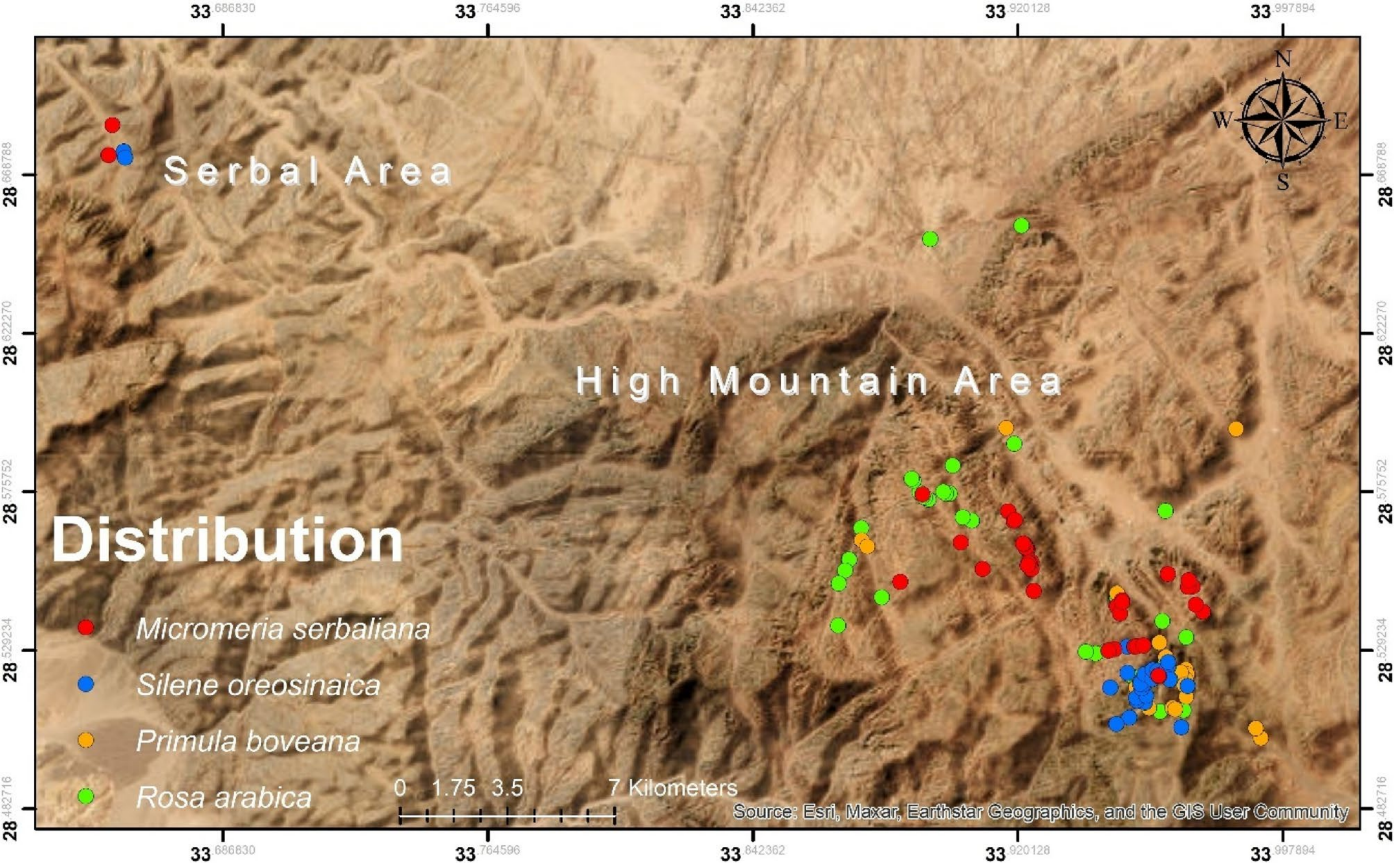
Recorded sub-populations of target species inside St. Catherine Protected Area

Vegetation analysis reveals that sites 8, 2, 7, and 3 exhibit the highest densities of *P. boveana*, *R. arabica*, *M. serbaliana*, and *S. oreosinaica*, respectively. The target species population shows clear fragmentation due to the area’s topographical features and natural barriers within the mountains (Fig. 5, S3). However, this study identified new areas for *Primula* and *Rosa*. Furthermore, a significant number of deceased and desiccated specimens of *P. boveana*, *M. serbaliana*, and *S. oreosinaica* were recorded, likely due to insufficient water.

### Habitat and ecology

All target species are situated within the confines of the SCPA, which encompasses mountainous ecosystems. The IUCN habitat classification scheme indicates that these species are found in rocky, mountainous, and desert regions. Despite the limited distribution and small population sizes of the target species, notable differences exist in their occupancy rates and frequency across microhabitats. During the field survey, it was observed that over 84% of the *P. boveana* population is confined to cliffs, whereas 53% and 35% of *R. arabica* are found in gorges and wadis, respectively. Over 45% of the *M. serbaliana* population is located on slopes, while over 75% of *S. oreosinaica* is situated on cliffs. The presence of *P. boveana* is confined to mountain cliffs nourished by melted snow and is prevalent in the moist soil surrounding wells and in safeguarded mountainous regions with year-round permanent water sources (Fig. S4 - supplementary materials).

**Fig. 6:**
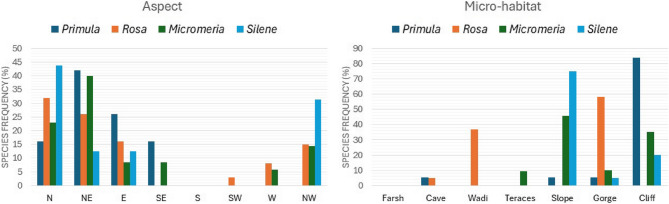
Target species distribution pattern among different microhabitat, and aspect (N: North, NE: Northeastern, E: East, SE: Southeastern, S: South, SW: Southwestern, W: West, and NW: Northeastern)

Examining topography as a significant factor influencing plant distribution, particularly in mountainous regions, revealed that the species of interest exhibited variations in their distribution relative to solar exposure (aspect). Specifically, *P. boveana* was predominantly located in the northeast and east directions, accounting for 44% and 25%, respectively, whereas *R. arabica* was primarily found on northern and northeastern slopes, representing 30% and 25% of its distribution. *Micromeria serbaliana* was observed on northeastern and northern slopes, accounting for 40% and 22%, respectively. In contrast, *S. oreosinaica* was identified on slopes facing northeast and northwest, with occurrences of 45% and 32%, respectively (Fig. 6).

Regarding the newly observed locations during this study, despite their presence north of the historically known geographical distribution for extended periods, we encountered them within the same previously inaccessible habitats, situated away from the conventional routes of prior surveys. Specifically, these locations, found north of the current distribution, hosted very small populations in water-rich cliffs for *Primula*, and rocky gorges for *Rosa*.

The physical heterogeneity in this region leads to climatic fluctuations, with the high elevation of Mount St. Catherine recognized as the coldest in the Sinai Peninsula. The average minimum temperature of the coldest month (2010–2024) is −6°C, and the average maximum temperature of the hottest month for the same period is 42°C. The average annual total precipitation for this arid region is roughly 45 mm (from 2010 to 2024), with some precipitation falling as snow. Nevertheless, there is considerable interannual fluctuation, potentially reaching up to 350 mm in any given year, primarily between October and June. The average yearly relative humidity is low at 40%, yet potential evaporation rates are exceedingly high, exceeding 18 mm/day in August.

Regarding the suitable range of soil properties, *P. boveana* is found in rocky soil with a texture composition of loamy (50%), loamy sand (35.5%), and sandy (14.5%). *R. arabica* is found in rocky soil characterized by a texture of loamy sand (41%), sandy (37.5%), and sandy loam (22.5%). *M. serbaliana* exhibits sandy to loamy sand textures, while *S. oreosinaica* displays a texture ranging from loamy sand to sandy. The target species are found in alkaline and non-saline to slightly saline soils with a pH range of 7.1 to 8.8 ± 0.46. These soils are defined by low water content and moderate levels of essential nutrients and cation-exchange capacity (CEC). The soil Electrical Conductivity (E.C. µS/cm) exhibited significant variation across different locations in SCPA, with values ranging from 104 to 1086. The same is observed in Total Dissolved Solids (T.D.S.) (Table S1).

Based on the differences in microhabitats, it was observed that the nature and quality of plant species accompanying the targeted species varied. For *P. boveana*, *Mentha longifolia* (100%), *Adiantum capillus-veneris* (100%), *Juncus rigidus* (80%), and *Hypericum sinaicum* (55%) are the most frequent species within the study area. In contrast, *Tanacetum sinaicum* (35%), *Achillea fragrantissima* (24.2%), *Phlomis aurea* (15%), *Mentha longifolia* (14.3%), *Zilla spinosa* (8%), *Juncus rigidus* (6.9%), *Stipa parviflora* (3.4%), *Echinops spinosus* L. (3.4%), and *Conyza bonariensis* (2.5%) are the most frequently associated species with *R. arabica*.

The circumstances differ for *M. serbaliana* and *S. oreosinaica*, which inhabit only mountain fractures and connections between adjacent rocks in sloped microhabitats. *Tanacetum sinaicum*, *Chiliadenus montanus*, *Pterocephalus sanctus*, and *Galium setaceum* are the species most associated with this microhabitat.

#### Threats

Despite being located within the SCPA (Protected Area), being subject to monitoring and evaluation programs, and with in situ conservation programs through rehabilitation underway, these species face numerous threats, both natural and anthropogenic. However, the newly discovered areas where *Primula boveana* occurs serve as indicators for assessing climate change, as its presence is associated with water availability. The primary natural threats identified include long-term drought, which has affected 100% of the sites within the small sub-population, alongside irregular and scarce rainfall patterns. Additionally, habitat fragmentation and the risk of flash floods contribute to significant issues, including an observed 15% loss due to uprooting.

*Rosa arabica* faces challenges not only from drought and limited rainfall but also from various detrimental human activities, such as overgrazing, harvesting for traditional medicine, and utilization as firewood and construction material for garden walls. Observations indicate that over 25% of the biomass of mature plants is harvested and utilized by humans. Furthermore, the germination of seeds and their success rates in their natural habitat present significant challenges to the propagation of these two species.

Alongside severe drought, *M. serbaliana* and *S. oreosinaica* face significant threats from overgrazing, potentially attributed to wild donkeys or domestic animals belonging to Bedouins residing near the high mountainous region. This activity eliminates seed content and reproductive structures, thereby decreasing the likelihood of seed production and dispersal (Table S2 & Figs. S5, and 6 - Supplementary materials).

### Extinction risk

The current field survey revealed discovery of new areas north of the previously known distribution in addition to the inclusion of the rehabilitated sites that have been carried out in 2021, resulting in a distinctive spatial change in both the Extent of Occurrence (EOO) and Area of Occupancy (AOO). Consequently, the conservation status of *Rosa arabica* has been relisted from Critically Endangered (CR) to Endangered (EN - B1ab (i, ii, iii, iv, v); C2a (i)). Specifically, the EOO increased from 47 km² before this study to 102 km², prompting this change in classification (Table 5, Fig. S7).

For the other three species, despite observed changes in their geographic range, population size, and threats, they did not meet the criteria to be reclassified from Critically Endangered to Endangered. Therefore, these targeted species remain classified as Critically Endangered (CR) according to the IUCN Petitions Committee criteria [79], consistent with the findings of Omar and El Gamal (2021a-f) [22–27]. Their geographical ranges are notably restricted, with the following recorded values: *P. boveana* (EOO: 72 km², AOO: 36 km² [B1ab (i, ii, iii, iv, v) + 2ab (i, ii, iii, iv, v)]), *R. arabica* (EOO: 88 km², AOO: 48 km² [B1ab (i, ii, iii, iv, v); C2a (i)]), *M. serbaliana* (EOO: 61 km², AOO: 16 km² [B1ab (ii, iii) + 2ab (ii, iii)]), and *S. oreosinaica* (EOO: 61 km², AOO: 16 km² [B1ab (ii, iii)]). As previously outlined, these populations are significantly small, highly fragmented, and experiencing a decline in habitat quality. Although the discovery of new areas has expanded their geographical distribution, these species are subject to fluctuations in population size, the number of individuals, and habitat quality. A decrease in the number of individuals has been observed in some sub-populations, while an increase has occurred in others, necessitating close monitoring by SCPA management.

**Table 5:** Recorded changes in IUCN red list conservation status for the target species

Scientific name	EOO (km^2^)		AOO (km^2^)		IUCN RL status	
	Before	After	Before	After	Before	After
*Primula boveana*	18.5	72.8	24	36	CR - B1ab (i, ii, iii, iv, v) + 2ab (i, ii, iii, iv, v)	CR - B1ab (i, ii, iii, iv, v) + 2ab (i, ii, iii, iv, v)
*Rosa arabica*	47	102	46	52	CR - B1ab (i, ii, iii, iv, v); C2a (i)	EN - B1ab (i, ii, iii, iv, v); C2a (i)
*Micromeria serbaliana*	68	88	40	48	CR - B1ab (ii, iii)	CR - B1ab (ii, iii)
*Silene oreosinaica*	53	61	12	16	CR - B1ab (ii, iii) + 2ab (ii, iii)	CR - B1ab (ii, iii) + 2ab (ii, iii)

### Species Distribution Model (SDM)

#### Model performance and major environmental factors

During this study, a total of 17 variables were selected based on multicollinearity and regression analysis, comprising 9 climatic, 3 topographic, and 5 edaphic factors. The target species exhibited slight variations in the number and type of environmental factors included in their respective models: 8 variables for *R. arabica* and 7 variables for each of *P. boveana*, *M. serbaliana*, and *S. oreosinaica* (Table 2).

The MaxEnt model demonstrated high success rates in predicting potentially suitable habitat, with Area Under the Curve (AUC) values for training data of 0.97 for *P. boveana*, 0.98 for *R. arabica*, 0.98 for *M. serbaliana*, and 0.97 for *S. oreosinaica*. Under the prevailing climatic conditions, the model showed strong predictive performance, as evidenced by AUC values exceeding 0.95. The True Skill Statistic (TSS) values for the current climatic conditions were also high: 0.92 for *P. boveana*, 0.91 for *R. arabica*, 0.89 for *M. serbaliana*, and 0.85 for *S. oreosinaica*. These results indicate that the MaxEnt models were reliable for predicting the potential geographical distribution areas of the target species in Egypt. Training, test AUC, and TSS values under current and future climatic scenarios (2050–2070) for each target species are presented in Table S3.

The internal jackknife test of factor importance for the MaxEnt model revealed that annual precipitation (Bio 12–47.9%), mean temperature of the wettest quarter (Bio 8–30%), elevation (8.3%), and mean temperature of the coldest quarter (Bio 11–7.5%) were the primary factors influencing the distribution of *P. boveana*. For *R. arabica*, the primary influencing factors were precipitation of the coldest quarter (Bio 19–86%), water content (6.6%), and mean diurnal range (Bio 2–4.6%). The distribution of *M. serbaliana* was primarily influenced by mean temperature of the wettest quarter (Bio 8–55.3%), annual precipitation (Bio 12–26.6%), isothermality (Bio 3–8.6%), and aspect (5.3%). Factors such as precipitation of the wettest month (Bio 13–55.3%), mean temperature of the wettest quarter (Bio 8–31.3%), and coarse content (6.2%) were identified as the primary controlling factors for *S. oreosinaica* (see Table 2).

According to the response curves of the leading environmental variables under the current conditions (Figs. S8–S11), the most suitable habitat conditions for the target species are identified as follows:

- *Primula boveana*: The probability of presence increases with increasing values of Bio 4, Bio 12, elevation, slope, clay content, and organic carbon content. Notably, the species was found in areas with annual precipitation (Bio 12) ranging from 53 to 105.00 mm, with optimal presence at 105 mm. Elevation ranged from 1760 to 2500 m above sea level (m a.s.l.), with an optimum at 2400 m a.s.l., and slope ranged between 5 and 24 degrees, with an optimum at 23 degrees. Organic carbon content ranged from 15.96 to 20.48% by weight (wt.), with an optimum at 20.4% wt., and pH ranged from 7.81 to 7.90, with an optimum at 7.9 (Details in Table S4).
- *R. arabica*: Its probability of presence increases with increasing values of Bio 2, Bio 12, Bio 19, elevation, organic carbon content, and water content. The species was observed in areas with an annual mean temperature (Bio 1) ranging from 13.5 to 16.1 °C, with optimal presence at 15 °C. Precipitation of the driest month (Bio 14) ranged from 0 to 1 mm, with optimal presence at 1 mm. Elevation ranged from 1400 to 2300 m a.s.l., with an optimum at 2000 m a.s.l., and slope ranged between 1 and 13 degrees, with an optimum at 4 degrees. The pH ranged from 7.81 to 7.90, with an optimum at 7.8, and organic carbon content ranged from 15.6 to 19.7%, with an optimum at 19%.
- *M. serbaliana*: The probability of presence increases with increasing values of Bio 12, clay content, elevation, and slope. Temperature annual range (Bio 7) ranged between 28.2 and 29.1 °C, with an optimum at 29 °C, and precipitation of the driest month (Bio 14) ranged from 0 to 1 mm, with optimal presence at 1 mm. Elevation ranged from 1800 to 2350 m a.s.l., with an optimum at 2300 m a.s.l., and slope ranged between 1 and 20 degrees, with an optimum at 20 degrees. Clay content ranged between 280 and 380 centimoles per kilogram (cmol/kg), with an optimum at 380 cmol/kg.
- *S. oreosinaica*: The probability of species presence increases with increasing values of Bio 12, Bio 13, elevation, slope, and water content. The species was found in areas with an annual mean temperature (Bio 1) ranging from 12.5 to 20 °C, with optimal presence at 13 °C, and maximum temperature of the warmest month (Bio 5) ranging from 26.9 to 33 °C, with an optimum at 26 °C. Mean temperature of the coldest quarter (Bio 11) ranged between 3.9 and 11.6 °C, with an optimum at 5 °C. Elevation ranged from 880 to 2500 m a.s.l., with an optimum at 2400 m a.s.l., and slope ranged between 4.5 and 20 degrees, with an optimum at 20 degrees.

#### Potential habitat suitability under current condition

**Fig. 7:**
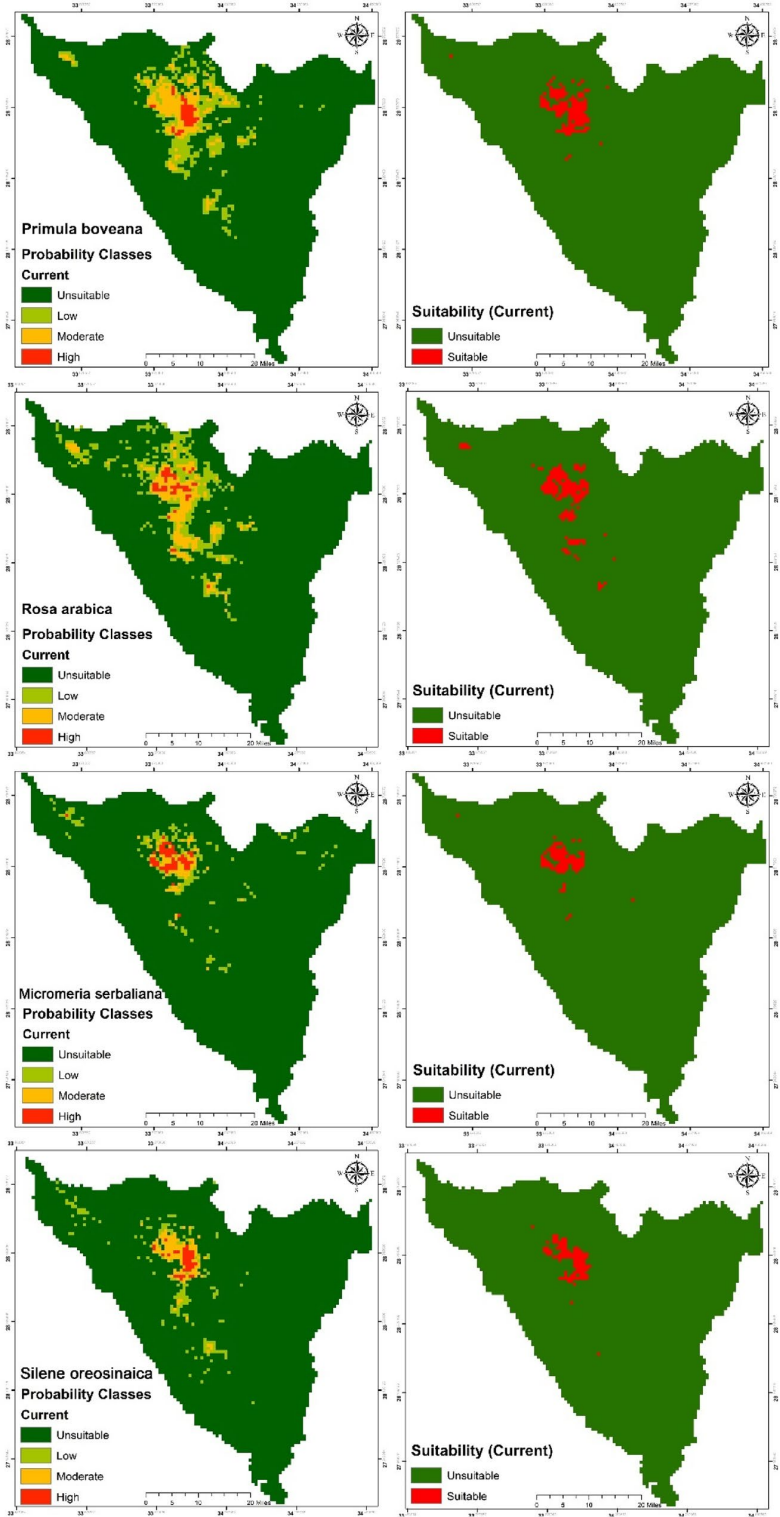
Map of the current potential habitat for target species; A = Probability, and b = Suitability

The findings indicate that the potential distribution of *P. boveana* encompasses an area of 500 km², representing 11.3% of the total SCPA area. This area comprises 36 km² (0.8%) classified as high probability (≥ 0.71), 167.2 km² (3.7%) as moderate probability (0.70–0.31), and 296.8 km² (6.7%) as low probability (0.30–0.11). The remaining area, totaling 3930 km² (88.7%), is categorized as unsuitable. The potential distribution of *R. arabica* encompasses an area of 533 km², representing 12.1% of the total SCPA area. This area is categorized into high probability (≥ 0.71) covering 37.4 km² (0.8%), moderate probability (0.70–0.31) encompassing 180.8 km² (4.1%), and low probability (0.30–0.11) accounting for 316.8 km² (7.1%); the remaining area, totaling 3897.4 km² (87.9%), is classified as unsuitable (Table 6; Fig. 7).

The estimated potential distribution of *M. serbaliana* encompasses an area of 232 km², representing 5.2% of the total SCPA area. This area is categorized into high probability (≥ 0.71) covering 48 km² (1.1%), moderate probability (0.70–0.31) at 57.6 km² (1.3%), and low probability (0.30–0.11) comprising 126.4 km² (2.9%); the remaining area, totaling 4198.4 km² (94.8%), is classified as unsuitable. The potential distribution of *S. oreosinaica* encompasses an area of 469 km², representing 5.4% of the total SCPA area. This area is categorized into 32.1 km² (0.7%) with high probability (≥ 0.71), 85.9 km² (1.9%) with moderate probability (0.70–0.31), and 121 km² (2.7%) with low probability (0.30–0.11); the remaining area is classified as unsuitable (4193.3 km², 94.6%) (Table 6; Fig. 7).

All targeted species exhibited high probability rates (≥ 0.71) within the boundaries of SCPA, particularly in the northwestern region (the high mountains and the Serbal areas), while demonstrating moderate to low probability presence in the central area for *S. oreosinaica* and *R. arabica*. The distribution is notably fragmented due to the presence of mountain barriers.

**Table 6:** Area and percentage of each habitat suitability categories for target species under current and future climatic scenarios

*Primula boveana*										
Ranks	Current	%	2050				2070			
			SSP 2.6	%	SSP 8.5	%	SSP 2.6	%	SSP 8.5	%
High	36	0.8	35.2	0.8	34.4	0.8	29.6	0.7	28.0	0.6
Moderate	167.2	3.7	180.8	4.1	163.2	3.7	193.6	4.4	188.8	4.3
Low	296.8	6.7	175.2	4.0	310.4	7.0	340.0	7.7	326.4	7.4
Unsuitable	3930.4	88.7	4039.2	91.2	3922.4	88.5	3867.2	87.3	3887.2	87.7
*Rosa arabica*										
Ranks	Current	%	2050				2070			
			SSP 2.6	%	SSP 8.5	%	SSP 2.6	%	SSP 8.5	%
High	37.4	0.8	36.6	0.8	41.1	0.9	38.1	0.9	42.6	1.0
Moderate	180.8	4.1	175.6	4.0	178.6	4.0	180.1	4.1	168.1	3.8
Low	316.8	7.1	316.8	7.1	310.8	7.0	321.3	7.2	300.4	6.8
Unsuitable	3897.4	87.9	3903.4	88.1	3901.9	88.0	3892.9	87.8	3921.3	88.5
*Micromeria serbaliana*										
Ranks	Current	%	2050				2070			
			SSP 2.6	%	SSP 8.5	%	SSP 2.6	%	SSP 8.5	%
High	48.0	1.1	52.8	1.2	48.8	1.1	55.2	1.2	44.0	1.0
Moderate	57.6	1.3	68.0	1.5	60.0	1.4	72.0	1.6	73.6	1.7
Low	126.4	2.9	150.4	3.4	125.6	2.8	162.4	3.7	143.2	3.2
Unsuitable	4198.4	94.8	4159.2	93.9	4196.0	94.7	4140.8	93.5	4169.6	94.1
*Silene oreosinaica*										
Ranks	Current	%	2050				2070			
			SSP 2.6	%	SSP 8.5	%	SSP 2.6	%	SSP 8.5	%
High	32.1	0.7	37.4	0.8	34.4	0.8	24.7	0.6	19.4	0.4
Moderate	85.9	1.9	101.6	2.3	83.7	1.9	100.9	2.3	106.1	2.4
Low	121.0	2.7	118.1	2.7	142.7	3.2	183.8	4.1	194.3	4.4
Unsuitable	4193.3	94.6	4175.4	94.2	4171.6	94.1	4123.0	93.0	4112.6	92.8

#### Potential habitat suitability changes under future conditions

The MaxEnt model indicated a potentially suitable habitat with high success rates, averaging over 0.97 for AUC (Training/Test) and over 0.8 for TSS values under future climatic scenarios, as shown in Table S3. Probability of occurrence areas and percentages (high, moderate, low, and unsuitable) for the entire SCPA region were extracted and are detailed in Table 6 and Fig. S12 across all climatic scenarios. Maps of the current and future potential suitable habitat for target species are presented in the supplementary materials (Figs. S13–S16).

**Fig. 8:**
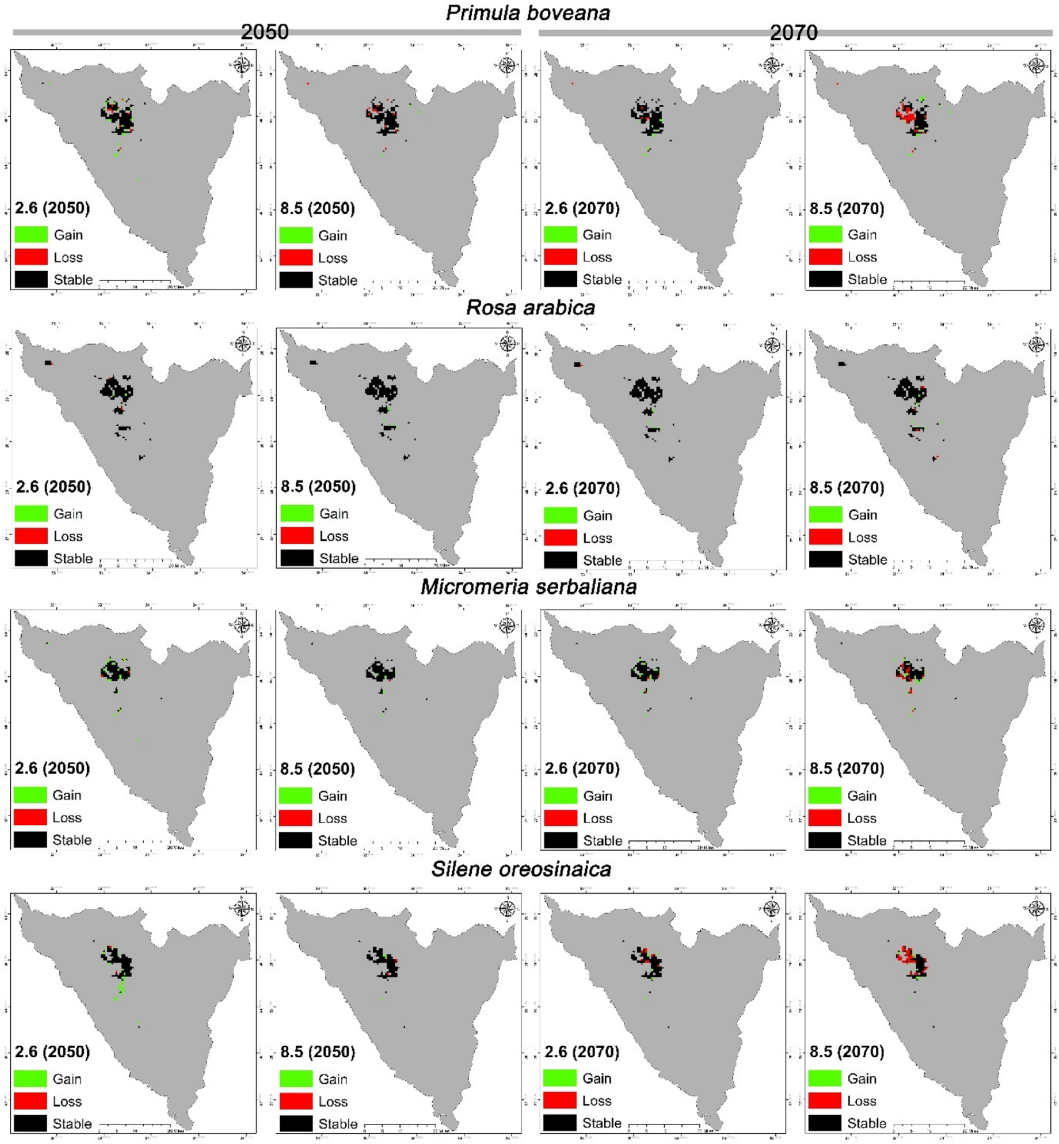
The Projection of the potential distribution changes maps of target species under future climate change scenarios. From top to down; *P. boveana*,* R. arabica*,* M. serbaliana* and *S. oreosinaica*

The spatial impacts of climate change were evaluated for the 2050 s and 2070 s under the SSP1 2.6 and SSP5 8.5 scenarios by analyzing regions with high suitability under both current and anticipated climate conditions. Data regarding areas gained, lost, and those that remained stable or unchanged were compiled (refer to Table 7; Fig. 8). *P. boveana* is projected to expand its suitable area by roughly 7.3 km² under the SSP1 2.6 scenario for 2050 and by 2.13 km² under the SSP5 8.5 scenario for the same year. Projections for 2070 indicate an expected gain of approximately 7.67 km² under SSP1 2.6 and 9.6 km² under SSP5 8.5. The results showed that this species will face a loss of suitable areas in the future, ranging from 7 to 32% of the areas currently suitable for its presence. Projected losses are 7.74 km², 9.66 km², 4.53 km², and 34.9 km² for the scenarios 2050 - SSP1 2.6, 2050 - SSP5 8.5, 2070 - SSP1 2.6, and 2070 - SSP5 8.5, respectively. Projected stability in suitable areas is expected to remain at approximately 92.8%, 91.1%, 95.8%, and 67.8% under the future scenarios for 2050 - SSP1 2.6, 2050 - SSP5 8.5, 2070 - SSP1 2.6, and 2070 - SSP5 8.5, respectively (Table 7).

*Rosa arabica* showed high stability in the future; it is projected to gain areas of approximately 1.8, 2.8, 2.8, and 4.4 km², while losing areas of 2.3, 0.3, 0.6, and 4.5 km². The species is expected to maintain very high stability in about 98%, 99.9%, 99.5%, and 96.2% of its current suitable area under the future scenarios for 2050 - SSP1 2.6, 2050 - SSP5 8.5, 2070 - SSP1 2.6, and 2070 - SSP5 8.5, respectively.

For *M. serbaliana* and *S. oreosinaica*, significant spatial changes in suitable habitats have been observed for the future. *M. serbaliana* is projected to lose approximately 0.7% of its suitable habitat while gaining about 6% by 2050, indicating an overall expansion. However, by 2070, its habitat is projected to shrink, with a loss of about 24% and a gain of only about 20%. In contrast, *S. oreosinaica* is projected to remain stable in 2050 but shrink significantly by 2070, with projected losses ranging from 2 to 41% of its current presence and gains between 2% and 23%.

**Table 7:** Areas, and percentages of gain, loss, and unchanged/stable categories

*Primula boveana*								
	2050				2070			
Category	Area 2.6 (km^2^)	%	Area 8.5 (km^2^)	%	Area 2.6 (km^2^)	%	Area 8.5 (km^2^)	%
Gain	7.29	6.7	2.13	1.96	7.67	7.05	9.61	8.83
Loss	7.74	7.12	9.66	8.88	4.53	4.16	34.99	32.16
Stable	101.06	92.88	99.14	91.12	104.27	95.84	73.81	67.84
*Rosa arabica*								
Gain	1.82	1.51	2.78	2.31	2.78	2.31	4.41	3.66
Loss	2.31	1.92	0.03	0.02	0.56	0.46	4.50	3.74
Stable	117.99	98.08	120.27	99.98	119.74	99.54	115.80	96.26
*Micromeria serbaliana*								
Gain	14.75	20.72	4.41	6.20	9.53	13.39	12.16	17.09
Loss	2.63	3.70	0.53	0.74	2.67	3.75	17.26	24.24
Stable	68.57	96.30	70.67	99.26	68.53	96.25	53.94	75.76
*Silene oreosinaica*								
Gain	17.77	23.32	2.25	2.95	4.36	5.73	7.33	9.62
Loss	1.92	2.52	1.98	2.60	7.44	9.76	31.60	41.46
Stable	74.30	97.48	74.23	97.40	68.78	90.24	44.61	58.54

Analysis of the spatial geographic variation in habitat suitability reveals distinct patterns of gain and loss. Our observations indicate that lost areas are predominantly confined to small, fragmented clusters situated within the peripheral and scattered regions of the high mountainous zones. Conversely, gained areas are projected to occur at higher elevations in the northern, central, and southern reaches of the current distribution. These newly acquired regions are characterized by mountainous terrain, consistent with the elevational preferences of the target species. Specifically, significant habitat gains are predicted in mountainous regions such as Catherine (2640 m), Ramghan (2415 m), Shatt (2427 m), Sabbah (2265 m), and Khalifa (2240 m) (Fig. 8). It is critical to emphasize that the four species under investigation are anticipated to differentially colonize these newly acquired mountainous regions, each according to their unique ecological niche and specific environmental requirements. *Primula* will primarily expand within its current range, acquiring interstitial areas. *Rosa*, conversely, will focus on gaining ground adjacent to its existing southern territories. *Micromeria* and *Silene* will stabilize their range expansion, primarily colonizing topographically similar inter-mountain zones within their current distribution in the Catherine, El Ahmar, and Galala Mountains.

## Discussion

Understanding the status and distribution of floral diversity is essential for developing effective conservation strategies, particularly for identifying endemic plant species unique to a region. This understanding represents a fundamental step in species conservation initiatives. This study provided details on the geographical distribution, population status, habitats, ecological characteristics, and major threats, while identifying the environmental factors influencing the distribution of the target species. The findings indicated that species distribution along an elevation gradient was affected by climatic factors, geographic location, historical context, and disturbances [124–127].

It was found that the EOO for *P. boveana*, *R. arabica*, *M. serbaliana*, and *S. oreosinaica* is 72.8 km² (280% increase), 102 km² (117% increase), 88.5 km² (30% increase), and 61 km² (15% increase), respectively, while the AOO is 36 km² (50% increase), 48 km² (4% increase), 52 km² (30% increase), and 16 km² (33% increase), respectively. As a result, the distribution and population count varied but remained within one to two main locations inside SCPA. *P. boveana* and *R. arabica* occur exclusively in the “High Mountains Area.” *M. serbaliana* and *S. oreosinaica* are distinctly located in two regions: the High Mountains Area and the Serbal Area, spanning east to west across the South Sinai area. However, despite the geographical changes, the results are still consistent with the findings of other previous studies [22–27, 29, 54, 56, 57, 59, 60, 66, 70, 76, 77, 128].

However, the reported number of endemic species in SCPA varies among different studies, ranging from 15 to 19 [87, 91]. It is worth noting that that in our field observations SCPA alone hosts 16 endemic plant species, accounting for 32.6% of Egypt’s total endemic species. The previously discussed high levels of endemism in these regions can be attributed to the geographic isolation of mountain ranges from adjacent lowlands, which serve as barriers to species dispersal. This isolation fosters specialization among species in unique high-altitude environments. According to Qian et al. (2024), “increased topographic variation may further enhance endemism by creating diverse habitats that promote isolation-driven specialization along ecological gradients” [129]. Gebrehiwot et al. (2019) also highlighted that “spatial variability and extreme microclimates often result in higher numbers of endemic plant species at elevated altitudes compared to lower elevations” [130].

The 2024–2025 field survey yielded crucial new data that directly influenced the IUCN Red List conservation status of one targeted species (*Rosa arabica*). By revealing previously unknown northern distribution areas, this discovery in addition to the inclusion of the rehabilitated sites that have been carried out in 2021 significantly increased both the Extent of Occurrence (EOO) from 47 km² to 102 km² and the Area of Occupancy (AOO) [23, 25]. Consequently, the species was reclassified from Critically Endangered (CR) to Endangered (EN B1ab(i, ii, iii, iv, v); C2a(i)). In contrast, despite observed changes in geographic range, population size, and threats for the other three surveyed species, these alterations did not meet the criteria necessary for reclassification. As a result, they remain listed as Critically Endangered (CR), consistent with prior assessments. The distinction between extinction risk categories, and even between purely endemic and near-endemic species, along with the potential for their status to change, hinges on updated geographical data [131, 132]. At the national level, a significant variation was observed between studies regarding the number of plant species endemic to Egypt, with figures ranging from 41 to 70 [87, 91, 133–135]. This variability depends on expanding knowledge of species’ distributions and the status of their habitats and threats [30, 131].

Alongside climatic and edaphic factors, the response curves demonstrate that topographic factors play a significant role in the distribution patterns of the target species. Observations indicate that the likelihood of species’ presence rises with altitude, whereas all species, except for *R. arabica*, are generally more abundant on steeper slopes. Field surveys and previous research [22–27, 29, 54, 56, 57, 59, 60, 66, 70, 76, 77, 128] corroborate that *P. boveana*, *M. serbaliana*, and *S. oreosinaica* prefer steep slopes or cliffs, while *R. arabica* is predominantly located in flat areas and wadis between gorges.

Our analysis indicates a future decline in the suitable habitat for the target species, though the magnitude of this reduction varies depending on the ecological characteristics of each species. While these species are projected to colonize new areas under future climate scenarios, the extent of these gains is consistently smaller than the anticipated losses in their current suitable ranges. This suggests a net contraction of habitat for all analyzed species. Specifically, *P. boveana* is expected to experience notable losses ranging from 7 to 32% of its current habitat, despite projected gains. *Rosa arabica* exhibits a contrasting pattern of high stability, with minimal losses and modest gains, resulting in a largely unchanged distribution. In contrast, *M. serbaliana* and *S. oreosinaica* are projected to face significant reductions in suitable habitat (reached to 41, and 23%, respectively), with losses potentially exceeding gains by a considerable margin, highlighting their vulnerability to future climate change. In agreement with many research, *Primula boveana*,* M. serbaliana*, and *S. oreosinaica*, with their specialized ecological needs concerning water availability, specific soil content, precipitation, and preference for particular topographical features like cliffs and steep slopes, are inherently more vulnerable to environmental changes than the more adaptable *Rosa arabica*; consequently, these species will likely experience a more significant decline due to their narrow habitat requirements being severely impacted by the increasing frequency and intensity of extreme weather events such as prolonged droughts and sudden floods, which their restricted distributions and small population sizes offer little resilience against [22–27, 30, 99, 136].

The target species, facing climate change pressures, is expected to seek refuge in higher altitudes, as evidenced by the potential gained areas in mountains like Catherine, Ramghan, Shatt, Sabbah, and Khalifa, which share similar elevations to their current habitat. However, the effectiveness of this adaptive strategy through migration will be significantly hampered by the existing fragmentation and isolation of suitable high-altitude habitats [137]. The lost areas, observed as small, scattered clusters in the peripheral high mountainous regions, highlight this challenge, indicating that the species’ ability to establish viable populations in these newly suitable, yet disconnected, locations will be severely limited and need future systematic field verification and monitoring.

These findings closely align with numerous studies indicating that climate change will significantly impact species distribution patterns. Extensive research has concluded that climate change will compel species to migrate to higher altitudes on mountain slopes [138, 139]. For instance, Manish et al. (2016) estimate that by 2050 and 2070, approximately 17% and 18% of endemic species in the Himalayas will lose their suitable habitats due to climate change [140]. This shift will prompt species with broader ranges to ascend to higher elevations, consequently influencing the distribution of endemic species at mountain summits as they face increased competition from more adaptable species. Furthermore, Di Musciano et al. (2021) discovered that rare high-altitude species in the Apuan Alps are likely to be endangered by competition from species migrating upward from lower altitudes due to climate change [141].

While ensemble modeling can often provide a more robust and accurate understanding of species distributions by integrating predictions from multiple models, this study demonstrates that a single MaxEnt model can still yield reliable results, particularly for species with restricted geographic ranges and specific environmental needs. Although all models inherently carry some level of uncertainty, the MaxEnt model’s success in predicting suitable habitats for the studied species underscores its value as a comprehensive tool for examining species-environment relationships [22–24, 142, 143], especially when considering the improved accuracy often observed for species with limited distributions and narrow environmental tolerances [114]. Given funding limitations for conservation efforts, the field data, informed by such SDM outputs, should guide the prioritization of conservation investments, highlighting the essential role of SDM in identifying potential future habitats in new regions.

### Conservation implications

This study underscored the paramount importance of establishing databases and baselines grounded in systematic surveys conducted at regular intervals to enable comparison and evaluation. Since 2010, we have been actively monitoring and tracking the environmental and spatial changes of these species, documenting trends, and assessing them according to the IUCN Red List criteria [22–27]. This ongoing monitoring has provided us with a 15-year database detailing the geographical distribution, habitat status, and threats. Our research recommended the utilization of such studies for continuous monitoring and status assessment to facilitate decisive and rapid conservation actions, including species reintroduction programs. Notably, we did not incorporate reintroduction sites into our current models, relying solely on wild individuals and populations, despite the successful outcomes of reintroduction trials observed after four years of cultivation.

Despite the encouraging discovery of new sites that led to a re-evaluation of its Extent of Occurrence (EOO) and Area of Occupancy (AOO), resulting in a change of *Rosa arabica*’s conservation status from Critically Endangered (CR) to Endangered (EN), this species and the other target species remains under persistent threats from both human activities, such as over-harvesting and habitat destruction, and natural factors like habitat fragmentation hindering seed dispersal and reducing adaptability [22–27, 71, 73, 84]. Consequently, urgent in situ and *ex situ* conservation practices are crucial, particularly focusing on areas identified as having high future environmental stability, to ensure the long-term survival and resilience of these target species.

The target species exhibits a global distribution within the study area (SCPA), where ongoing monitoring and evaluation plans are implemented throughout the year. The SCPA management team conducts educational and awareness initiatives for the local community, stakeholders, and regional partners. This research has established a population size baseline, facilitating comparisons with future population assessments and potential IUCN Red List updates under criteria A or C. Continuous monitoring of population and habitat trends, along with the fluctuations identified in this study, is strongly advised. Seed collection and storage are crucial for preserving genetic material in national and international seed banks, including those at Kew. Valderrábano et al. (2018) and Omar and Elgamal (2021 a-f) emphasize the urgent need to enhance both in-situ and *ex-situ* conservation initiatives for threatened species [22–27, 63, 91, 133–135]. These efforts should be accompanied by focused management, recovery, and reintroduction strategies at both species and population levels, particularly in the high-suitability areas identified in this research, while considering potential future spatial changes. Encouraging community involvement and participatory methods will improve the understanding of traditional values and practices, thereby facilitating effective plant conservation and reducing pressures on wild plant populations.

The study, while producing accurate models that align with species range maps, is not without limitations. This research relied solely on a single algorithm (Maxent) to directly model the probability of occurrence, which can be susceptible to the selection of background data. Although well-tuned individual models, including Maxent, can still yield comparable or even superior results in certain instances, ensemble modeling in species distribution modeling (SDM) involves integrating the predictions of multiple distinct SDM algorithms (which may include Maxent as one of the base models) or variations of the same algorithm. Research indicates that ensemble approaches can sometimes outperform single algorithms like Maxent by capitalizing on the strengths of diverse modeling techniques and mitigating uncertainty. This research incorporated various environmental factors, including climate, topography, and soil characteristics; however, the inclusion of additional factors could enhance the models. Integrating more variables, such as development, species interactions, dispersal patterns, land use, and demographic data, would lead to more accurate predictions, necessitating further ecological and conservation investigations. Despite numerous field studies conducted on various endemic plant species in Egypt, a significant gap persists in our understanding of the geographic distribution of threatened species, their ecological and population dynamics, habitat trends, threats, and conservation priorities. The lack of comprehensive data hinders the formulation and implementation of effective conservation strategies both inside and outside protected areas.

## Conclusion

This research investigated the effects of anticipated climate change on the geographical distribution of four critically endangered plant species located in the highest mountain ranges of South Sinai. To maximize the effectiveness of future conservation strategies for these threatened species, a combination of methodologies was employed, integrating Species Distribution Models (SDMs) with the IUCN Red List. Although the data gathered in this study is substantial, it is evident that further efforts are necessary for the conservation program. It is advisable to utilize the findings from this research, including tables and figures, as foundational elements for the formulation of effective conservation initiatives, both in situ and ex situ. The field investigations underscored the anticipated spatial alterations (losses, gains, and stability) resulting from climate change in areas of high suitability. The significance of conserving these species is emphasized through in situ recovery and reintroduction efforts, as well as ex situ seed collection and storage in national seed banks. These actions are crucial in light of the threats posed by grazing, drought, potential over-collection for scientific purposes, and habitat fragmentation. Furthermore, continuous monitoring through systematic surveys at regular time intervals is essential to stay updated on the existing ecological and conservation status and to adapt management strategies accordingly. Fostering environmental awareness among stakeholders, including local communities, partners, universities, and research institutions, regarding the necessity of protecting endemic species is of paramount importance.

## Supplementary Information

Supplementary Material 1.

## Data Availability

The datasets used and/or analysed during the current study are available from the corresponding author on reasonable request.

## References

[1] Lewis SL, Maslin MA. Defining the anthropocene. Nature. 2015;519(7542):171–80. 10.1038/nature14258. 25762280 10.1038/nature14258

[2] Barnosky AD, Matzke N, Tomiya S, Wogan GOU, Swartz B, Quental TB, et al. Has the earth’s sixth mass extinction already arrived? Nature. 2011;471(7336):51–7. 10.1038/nature09678. 21368823 10.1038/nature09678

[3] Heller NE, Zavaleta ES. Biodiversity management in the face of climate change: A review of 22 years of recommendations. Biol Conserv. 2008;142(1):14–32. 10.1016/j.biocon.2008.10.006.

[4] Pereira HM, Leadley PW, Proença V, Alkemade R, Scharlemann JPW, Fernandez-Manjarrés JF, et al. Scenarios for global biodiversity in the 21st century. Science. 2010;330(6010):1496–501. 10.1126/science.1196624. 20978282 10.1126/science.1196624

[5] Thomas CD, Cameron A, Green RE, Bakkenes M, Beaumont LJ, Collingham YC, et al. Extinction risk from climate change. Nature. 2004;427(6970):145–8. 10.1038/nature02121. 14712274 10.1038/nature02121

[6] Williams SE, Shoo LP, Isaac JL, Hoffmann AA, Langham G. Towards an integrated framework for assessing the vulnerability of species to climate change. PLoS Biol. 2008;6(12):e325. 10.1371/journal.pbio.0060325. 19108608 10.1371/journal.pbio.0060325 PMC2605927

[7] Post E. Ecology of Climate Change: The Importance of Biotic Interactions. Princeton University Press; 2013. 10.1515/9781400846139.

[8] Brook B, Sodhi N, Bradshaw C. Synergies among extinction drivers under global change. Trends Ecol Evol. 2008;23(8):453–60. 10.1016/j.tree.2008.03.011. 18582986 10.1016/j.tree.2008.03.011

[9] Abreu-Jardim TPF, Jardim L, Ballesteros-Mejia L, Maciel NM, Collevatti RG. Predicting impacts of global Climatic change oTn genetic and phylogeographical diversity of a Neotropical treefrog. Divers Distrib. 2021;27(8):1519–35. 10.1111/ddi.13299.

[10] Shi N, Naudiyal N, Wang J, Gaire NP, Wu Y, Wei Y, et al. Assessing the impact of climate change on potential distribution of *Meconopsis punicea* and its influence on ecosystem services supply in the southeastern margin of Qinghai-Tibet Plateau. Front Plant Sci. 2022;12. 10.3389/fpls.2021.830119. 10.3389/fpls.2021.830119 PMC8792861 35095992

[11] Wilson RJ, Gutiérrez D, Gutiérrez J, Martínez D, Agudo R, Monserrat VJ. Changes to the elevational limits and extent of species ranges associated with climate change. Ecol Lett. 2005;8(11):1138–46. 10.1111/j.1461-0248.2005.00824.x. 21352437 10.1111/j.1461-0248.2005.00824.x

[12] Parmesan C. Ecological and evolutionary responses to recent climate change. Annu Rev Ecol Evol Syst. 2006;37(1):637–69. 10.1146/annurev.ecolsys.37.091305.110100.

[13] Pauli H, Gottfried M, Dirnböck T, Dullinger S, Grabherr G. Assessing the long-term dynamics of endemic plants at summit habitats. In: Ecological studies. 2003. pp. 195–207. 10.1007/978-3-642-18967-8_9.

[14] La Sorte FA, Jetz W. Projected range contractions of montane biodiversity under global warming. Proc R Soc B Biol Sci. 2010;277(1699):3401–10. 10.1098/rspb.2010.0612. 10.1098/rspb.2010.0612 PMC2982223 20534610

[15] Pounds JA, Crump ML. Amphibian declines and climate disturbance: the case of the golden Toad and the harlequin frog. Conserv Biol. 1994;8(1):72–85. 10.1046/j.1523-1739.1994.08010072.x.

[16] Dirnböck T, Essl F, Rabitsch W. Disproportional risk for habitat loss of high-altitude endemic species under climate change. Glob Change Biol. 2010;17(2):990–6. 10.1111/j.1365-2486.2010.02266.x.

[17] Urban MC. Accelerating extinction risk from climate change. Science. 2015;348(6234):571–3. 10.1126/science.aaa4984. 25931559 10.1126/science.aaa4984

[18] Bonebrake TC, Brown CJ, Bell JD, Blanchard JL, Chauvenet A, Champion C, et al. Managing consequences of climate-driven species redistribution requires integration of ecology, conservation and social science. Biol Reviews/Biological Reviews Camb Philosophical Soc. 2017;93(1):284–305. 10.1111/brv.12344. 10.1111/brv.12344 28568902

[19] Lenoir J, Svenning J-C. Climate‐related range shifts– a global multidimensional synthesis and new research directions. Ecography. 2014;38(1):15–28. 10.1111/ecog.00967.

[20] Hickler T, Vohland K, Feehan J, Miller PA, Smith B, Costa L, et al. Projecting the future distribution of European potential natural vegetation zones with a generalized, tree species-based dynamic vegetation model. Glob Ecol Biogeogr. 2011;21(1):50–63. 10.1111/j.1466-8238.2010.00613.x.

[21] Hughes C, Eastwood R. Island radiation on a continental scale: exceptional rates of plant diversification after uplift of the Andes. Proc Natl Acad Sci. 2006;103(27):10334–9. 10.1073/pnas.0601928103. 16801546 10.1073/pnas.0601928103 PMC1502458

[22] Omar K, Elgamal I. IUCN red list and species distribution models as tools for the conservation of poorly known species: a case study of endemic plants Micromeria Serbaliana and Veronica kaiseri in South sinai, Egypt. Kew Bull. 2021;76(3):477–96. 10.1007/s12225-021-09953-4.

[23] Omar K, Elgamal I. Can we save critically endangered relict endemic plant species? A case study of *Primula Boveana* decne ex Duby in Egypt. J Nat Conserv. 2021;61:126005. 10.1016/j.jnc.2021.126005.

[24] Omar K, Elgamal I. Assess the extinction risk of mountain endemic plants in Egypt under the current Climatic condition: A case study of endemic *Silene* species. Eur J Biology Biotechnol. 2021;2(5):34–47. 10.24018/ejbio.2021.2.5.261.

[25] Omar K, Elgamal I. Conservation of challenging endemic plant species at high risk of extinction in arid mountain ecosystems: a case study of *Rosa arabica* Crép. in Egypt. J Mountain Sci. 2021;18(10):2698–721. 10.1007/s11629-021-6750-2.

[26] Omar K, Elgamal I. Micromeria serbaliana. The IUCN Red List of Threatened Species 2021:e.T184589270A184589325. Available from: 10.2305/iucn.uk.2021-2.rlts.t184589270a184589325.en.

[27] Omar K, Elgamal I. Silene oreosinaica. The IUCN Red List of Threatened Species 2021:e.T184589013A184589032. Available from: 10.2305/IUCN.UK.2021-2.RLTS.T184589013A184589032.en.

[28] Batanouny KH. Man’s impact on vegetation. In: Holzner W, Werger MJA, Ikusima I, editors. Human impact on desert vegetation. The Hague: Junk; 1983. pp. 139–49.

[29] Omar K, Mohammed AA, Nagi A, El Gamal I, Elmarakby A, Shalouf A et al. Conservation challenges inside protected areas of Egypt: Part II: St. Katherine Protectorate - Conservation status Assessment of some threatened plant species. Report to Nature Conservation Sector, Ministry of Environment - Strengthening protected area financing. Cairo, Egypt; 2015. https://www.researchgate.net/publication/328416406_Conservation_challenges_inside_protected_areas_of_Egypt-part_II-St_Katherine_Protectorate_Conservation_Status_Assessment_of_some_threatened_plant_species.

[30] Omar K. Impacts of climate change on the distribution of target species of fauna and flora in. Egypt: report for formulation & advancement of the National adaptation plan process project (NAP). Cairo, Egypt: Ministry of Environment, UNDP; 2025.

[31] Moustafa AA, Zaghloul MS, El-Wahab RA, Shaker M. Evaluation of plant diversity and endemism in saint Catherine protectorate, South sinai, Egypt. Egypt J Bot. 2001;41(1):121–39. https://www.academia.edu/11535746/Evaluation_of_plant_diversity_and_endemism_in_Saint_Catherine_Protectorate_South_Sinai_Egypt_Egyptian.

[32] Shabana H, Moursy MM, Omar K. Survey of the flora enclosures of st. Katherine protectorate: In-situ conservation. Plant conservation and monitoring programme report, saint Katherine protectorate. Cairo, Egypt: Ministry of Environment; 2011.

[33] Zhang C, Willis CG, Klein JA, Ma Z, Li J, Zhou H, et al. Recovery of plant species diversity during long-term experimental warming of a species-rich alpine meadow community on the Qinghai-Tibet plateau. Biol Conserv. 2017;213:218–24. 10.1016/j.biocon.2017.07.019.

[34] Li YH, Zhang LJ, Zhu WB, Zhang JJ, Xu SB, Zhu LQ. Changes of *Taxus chinensis* var. Mairei habitat distribution under global climate change. J Nat Resour. 2021;36(3):783–92. 10.31497/zrzyxb.20210318.

[35] Dillon ME, Wang G, Huey RB. Global metabolic impacts of recent climate warming. Nature. 2010;467(7316):704–6. 10.1038/nature09407. 20930843 10.1038/nature09407

[36] Dawson TP, Jackson ST, House JI, Prentice IC, Mace GM. Beyond predictions: biodiversity conservation in a changing climate. Science. 2011;332(6025):53–8. 10.1126/science.1200303. 21454781 10.1126/science.1200303

[37] McMahon SM, Harrison SP, Armbruster WS, Bartlein PJ, Beale CM, Edwards ME, et al. Improving assessment and modelling of climate change impacts on global terrestrial biodiversity. Trends Ecol Evol. 2011;26(5):249–59. 10.1016/j.tree.2011.02.012. 21474198 10.1016/j.tree.2011.02.012

[38] Parmesan C, Duarte C, Poloczanska E, Richardson AJ, Singer MC. Overstretching attribution. Nat Clim Change. 2011;1(1):2–4. 10.1038/nclimate1056.

[39] Omar K. A report on steps for determining impacts of climate change on the distribution of target species of fauna in Egypt using species distribution models (SDMS). Amman, Egypt: IUCN Regional Office for West Asia and Ottawa, Canada: Global Affairs Canada;; 2023. p. 145.

[40] Brooks R, M’Lot M, McLachlan SM. Pitfalls to avoid when linking traditional and scientific knowledge. In: Oakes JE, Riewe RR, editors. Climate change: linking traditional and scientific knowledge. Winnipeg, Canada: Aboriginal Issues Press, University of Manitoba; 2006. pp. 13–20.

[41] International Union for Conservation of Nature. IUCN red list categories and criteria: version 3.1. 2nd ed. Gland, Switzerland and Cambridge, UK: IUCN; 2012. https://portals.iucn.org/library/node/10315.

[42] Mace GM, Collar NJ, Gaston KJ, Hilton-Taylor C, Akçakaya HR, Leader‐Williams N, et al. Quantification of extinction risk: iucn’s system for classifying threatened species. Conserv Biol. 2008;22(6):1424–42. 10.1111/j.1523-1739.2008.01044.x. 18847444 10.1111/j.1523-1739.2008.01044.x

[43] Vié J, Hilton-Taylor C, Pollock C, et al. The IUCN red list: a key conservation tool. The 2008 review of the IUCN red list of threatened species. Gland, Switzerland: IUCN; 2008. https://www.iucn.org/sites/dev/files/import/downloads/the_iucn_red_list_a_key_conservation_tool_1.pdf.

[44] Cassini MH. Ranking threats using species distribution models in the IUCN red list assessment process. Biodivers Conserv. 2011;20(14):3689–92. 10.1007/s10531-011-0126-9.

[45] Elith J, Graham CH, Anderson RP, Dudík M, Ferrier S, Guisan A, et al. Novel methods improve prediction of species’ distributions from occurrence data. Ecography. 2006;29(2):129–51. 10.1111/j.2006.0906-7590.04596.x.

[46] Elith J, Leathwick JR. Species distribution models: Ecological explanation and prediction across space and time. Ann Rev Ecol Evol Syst. 2009;40(1):677–97. 10.1146/annurev.ecolsys.110308.120159.

[47] Ramirez-Villegas J, Cuesta F, Devenish C, Peralvo M, Jarvis A, Arnillas CA. Using species distribution models for designing conservation strategies of tropical Andean biodiversity under climate change. J Nat Conserv. 2014;22(5):391–404. 10.1016/j.jnc.2014.03.007.

[48] Wang HH, Wonkka CL, Treglia ML, Grant WE, Smeins FE, Rogers WE. Species distribution modelling for conservation of an endangered endemic Orchid. AoB Plants. 2015;7. 10.1093/aobpla/plv039.. 10.1093/aobpla/plv039 PMC4463238 25900746

[49] Zangiabadi S, Zaremaivan H, Brotons Li, Mostafavi H, Ranjbar H. Using Climatic variables alone overestimate climate change impacts on predicting distribution of an endemic species. PLoS ONE. 2021;16(9):e0256918. 10.1371/journal.pone.0256918. 34473770 10.1371/journal.pone.0256918 PMC8412407

[50] Qazi AW, Saqib Z, Zaman-Ul-Haq M. Trends in species distribution modelling in context of rare and endemic plants: A systematic review. Ecol Processes. 2022;11(1):1–11. 10.1186/s13717-022-00384-y.

[51] Hunter-Ayad J, Ohlemüller R, Recio MR, Seddon PJ. Reintroduction modelling: A guide to choosing and combining models for species reintroductions. J Appl Ecol. 2020;57(7):1233–43. 10.1111/1365-2664.13629.

[52] Moustafa ARA, Klopatek JM. Vegetation and landforms of the saint Catherine area, Southern sinai, Egypt. J Arid Environ. 1995;30(4):385–95. 10.1006/jare.1995.0033.

[53] Grainger J. People are living in the park’. Linking biodiversity conservation to community development in the middle East region: a case study from the saint Katherine protectorate, Southern Sinai. J Arid Environ. 2003;54(1):29–38. 10.1006/jare.2001.0894.

[54] Omar K. Vegetation, soil and grazing analysis in saint Katherine protectorate, South sinai, Egypt. NeBIO. 2013;3(2):80–92.

[55] Omar K, Elgamal I. Reproductive and germination ecology of Sinai primrose, *Primula Boveana* decne. Ex Duby. J Glob Biosci. 2014;3(4):694-707. ISSN 2320– 1355.

[56] Fayed AA, El-Garf IA, Abdel-Khalik KN, Osman AK. Floristic survey of the mountainous region of South Sinai, St Katherine’s Protectorate, Medicinal Plants Conservation Project. Egypt; 2004. pp 146. local report.

[57] Shaltout K, Al-Sodany Y, Heneidy S, Marie A, Eid E, Hatim M et al. Floristic survey of the mountainous southern Sinai: Saint Katharine Protectorate. Medicinal Plants Conservation Project. 2004. Available from: https://www.academia.edu/20025490/Floristic_Survey_of_the_Mountainous_Southern_Sinai_Saint_Katharine_Protectorate.

[58] Khedr AH, El-Katony T, Saad-Allah K, Ahmed F, Kashlana M. Niche differentiation of two congeneric *Phlomis* species in Egypt. Sci J Damietta Fac Sci. 2020;10(1):45–57. 10.21608/sjdfs.2020.194999.

[59] Omar KA. Ecological and Climatic attribute analysis for Egyptian *Hypericum Sinaicum*. Am J Life Sci. 2014;2(6):369. 10.11648/j.ajls.20140206.17.

[60] Omar K, IUCN Red List of Threatened Species 2014. *Primula boveana*. Available from: 10.2305/iucn.uk.2014-3.rlts.t163968a1015883.en.

[61] Richards J. *Primula*. *Pavilion Books. *2014. pp 156. ISPN 9781849942416, 156. https://books.google.com.eg/books?id=pxqUEAAAQBAJ.

[62] Radford EA, Catullo G, Montmollin B, editors. Important plant areas of the South and East Mediterranean Region: Priority sites for conservation. Gland, Switzerland and Malaga, Spain: IUCN; 2011. Available from: https://portals.iucn.org/library/sites/library/files/documents/2011-014.pdf.

[63] Valderrábano M, Gil T, Heywood V, de Montmollin B, editors. Conserving wild plants in the south and east Mediterranean region. Gland, Switzerland: Union Internationale pour la Conservation de la Nature; 2018. Available from: 10.2305/IUCN.CH.2018.21.en.

[64] El Hadidi MN. *Flora Aegyptiaca*. Vol. 1, part 1. Cairo: The Palm Press & Cairo University, Herbarium; 2000.

[65] Moustafa AA, Zaghloul MS, Mansour SR, Al Sharkawy DH, Alotaibi M. Long-term monitoring of *Rosa Arabica* populations as a threatened species in South sinai, Egypt. J Biodivers Endanger Species. 2017;5:1–8. 10.4172/2332-2543.1000197.

[66] Omar K. *Rosa arabica*. The IUCN Red List of Threatened Species. 2017;e.T84120072A84120074. Available from: 10.2305/IUCN.UK.2017-3.RLTS.T84120072A84120074.en.

[67] Täckholm V. Students’ flora of Egypt. Cairo: Cairo University; 1974.

[68] Boulos L. Flora of Egypt. Volume 1. Cairo, Egypt: Al Hadara Publishing; 1999.

[69] Abdullah A, Megahed K, El Mawi M, et al. Conservation and sustainable use of medicinal and wild plants in saint Katherine protectorate in Egypt. Cairo: Dar El Kotub; 2012. (Published by The Medicinal Plants Conservation Project).

[70] Moustafa AA, Abd El-Wahab RH, Zaghloul MS, El-Rayes AA. Botanical survey of saint Catherine protectorate. Final report. saint Catherine protectorate.Development project. Cairo: Egyptian Environmental Affairs Agency (EEAA); 1998.

[71] Assi R. Medicinal Plants Threat Analysis and Threat Reduction. Conservation and Sustainable Use of Medicinal Plants in Arid and Semi-Arid Ecosystems Project; UNDP/GEF report. 2007. pp 140.

[72] Grainger J, Gilbert F. Around the sacred mountain: the St Katherine protectorate in South sinai, egypt. Protected landscapes and cultural and spiritual values. the Series Values of Protected Landscapes and Seascapes. Volume 2. Gland, Switzerland: IUCN, GTZ, and Obra Social de Caixa Catalunya; 2008. p. 21.

[73] Mosallam HAM. Assessment of target species in saint Katherine protectorate, sinai, Egypt. J Appl Sci Res. 2007;3(6):456–69.

[74] Zaghloul MS, Hamrick JL, Moustafa AA, Kamel WM, El-Ghareeb R. Genetic diversity within and among Sinai populations of three *Ballota* species (Lamiaceae). J Hered. 2006;97(1):45–54. 10.1093/jhered/esj008. 16407527 10.1093/jhered/esj008

[75] Khedr AH. Regional patterns of rarity and life history elements in the flora of Egypt. Taeckholmia/Taeckholmia (Online). 2006;26(1):141–60. Available from: 10.21608/taec.2006.12290.

[76] Khedr A. Microhabitats supporting endemic plants in Sinai. In: Elkhouly A, Negm A, editors. Management and development of agricultural and natural resources in egypt’s desert. Switzerland: Springer Nature; 2021. pp. 369–81.

[77] Omar K. Eco-geographical studies on Nepeta septemcrenata in saint Katherine protectorate, South sinai, Egypt. Cairo: Al-Azhar University; 2010.

[78] Cox G. Laboratory manual of general ecology. 6th ed. Dubuque, Iowa: William C. Brown; 1990. p. 143.

[79] IUCN Standards and Petitions Committee. Guidelines for Using the IUCN Red List Categories and Criteria. Version 16. Prepared by the Standards and Petitions Committee. 2024. Available from: https://www.iucnredlist.org/documents/RedListGuidelines.pdf.

[80] Allen SE, Grimshaw HM, Parkinson JA, Quarmby C, Roberts JD. Chemical analysis of ecological materials. Oxford and London: Blackwell Scientific; 1974.

[81] Jackson ML. Soil chemical analysis. New Delhi: Prentice Hall of India Pvt. Ltd.; 1967. p. 498.

[82] Buurman P, Lagen E, Velthorst E. Manual for soil and water analysis. Leiden: Backhuys; 1996. p. 330.

[83] Braun-Blanquet J. Plant Sociology. Translated by Fuller GD, Conard HS. New York: McGraw-Hill Book Co. Inc.; 1964. 865 p.

[84] Khafagi O, Hatab EE, Omar K. Ecological niche modeling as a tool for conservation planning: suitable habitat for *Hypericum Sinaicum* in South sinai, Egypt. Univ J Environ Res Technol. 2013;2(6):515–24.

[85] Li Y, Zhang XW, Fang YM. Predicting the impact of global warming on the geographical distribution pattern of *Quercus variabilis* in China. Chin J Appl Ecol. 2014;25:3381–9. 25876385

[86] Miao J, Wang Y, Wang L, et al. Prediction of potential geographical distribution pattern change for *Castanopsis sclerophylla* on maxent. J Nanjing Univ (Nat Sci Ed). 2021;45:193–8.

[87] Boulos L. Flora of Egypt Checklist. Revised Annotated Edition. Cairo: Al-Hadara Publishing; 2009. 410 p.

[88] El-Demerdash M. Medicinal Plants of Egypt. In: Development of Plant-Based Medicines: Conservation, Efficacy and Safety. 2001. pp. 69–93.

[89] Abd El-Wahab R, Zaghloul M, Moustafa A. Conservation of medicinal plants in Saint Catherine Protectorate, South Sinai, Egypt: I. Evaluation of ecological status and human impact. In: Proceedings of the First International Conference on Strategy of Egyptian Herbaria; 2004 Mar 9–11; Mubarak City for Scientific Research & Technology Applications. 2004. pp. 231–251.

[90] Shaltout KH, Al-Sodany YM, Eid EM, Heneidy SZ, Taher MA. Vegetation diversity along the altitudinal and environmental gradients in the main wadi beds in the mountainous region of South Sinai, Egypt. J Mountain Sci. 2020;17(10):2447–58. Available from: 10.1007/s11629-020-6153-9.

[91] Hosni H, Hosny A, Shamso E, Hamdy R. Endemic and near-endemic taxa in the flora of Egypt. Egypt J Bot. 2013;53(2):357–83.

[92] Shaltout KH, Eid E. Important plant areas in egypt: with emphasis on the mediterranean region. Saarbrücken: Lambert Academic Publishing; 2016. p. 190.

[93] Murienne J, Guilbert E, Grandcolas P. Species’ diversity in the New Caledonian endemic genera Cephalidiosus and Nobarnus (Insecta: Heteroptera: Tingidae), an approach using phylogeny and species’ distribution modelling. Biol J Linn Soc. 2009;97(1):177–84. 10.1111/j.1095-8312.2008.01184.x.

[94] Boucher O, Servonnat J, Albright AL, Aumont O, Balkanski Y, Bastrikov V, et al. Presentation and evaluation of the IPSL-CM6A-LR climate model. J Adv Model Earth Syst. 2020;12:e2019MS002010. 10.1029/2019MS002010.

[95] Paul S, Lata S, Barman T. Habitat distribution modeling of the Pinus gerardiana under projected climate change in the North-Western himalaya, India. Landscape Ecol Eng. 2023;19(4):647–60.

[96] Acarer A. Role of climate change on Oriental Spruce (Picea orientalis L.): modeling and mapping. BioResources. 2024;19(2):3845.

[97] Sarikaya AG, Uzun A. Modeling the effects of climate change on the current and future potential distribution of Berberis vulgaris L. with machine learning. Sustainability. 2024;16(3):1230.

[98] Örücü ÖK, Azadi H, Arslan ES, Aksoy K, Choobchian Ö, Nooghabi S, S. N., Stefanie H. I. Predicting the distribution of European hop hornbeam: application of maxent algorithm and Climatic suitability models. Eur J for Res. 2023;142(3):579–91.

[99] Parveen S, Kaur S, Baishya R, Goel S. Predicting the potential suitable habitats of genus Nymphaea in India using maxent modeling. Environ Monit Assess. 2022;194(12):853. 36203117 10.1007/s10661-022-10524-8

[100] Li Y, Huang B, Tan C, Zhang X, Cherubini F, Rust HW. Investigating the global and regional response of drought to idealized deforestation using multiple global climate models. Hydrol Earth Syst Sci. 2025;29(6):1637–58.

[101] Abdel-Dayem M S, Al Dhafer H M, Soliman A M, Al Ansi A N, El-Sonbati S A, Ishag AA, Mohamed A, Soliman M. Climate change and geographical distribution projections for major leaf beetles (Coleoptera: Chrysomelidae) in Saudi Arabia. J Econ Entomol. 2025;118(2):600-13.10.1093/jee/toaf046. 10.1093/jee/toaf046 40037784

[102] Khedr AA, Serag MA, Elbaroughy RF, Abo Elagras HA. Modelling current and future distribution of some invasive weeds at local and global scales under Climatic change. Sci J Damietta Fac Sci. 2024;14(1):28–39.

[103] Zhao S, Zhang M, Wu Y, Guo E, Wang Y, Cui S, Kolerski T. Response of gross primary productivity (GPP) of the desert steppe ecosystem in the Northern foothills of Yinshan mountain to extreme climate. Land. 2025;14(4):884.

[104] Hengl T, de Jesus JM, MacMillan RA, Batjes NH, Heuvelink GBM, Ribeiro E, et al. SoilGrids1km—Global soil information based on automated mapping. PLoS ONE. 2014;9(8):e105992. 10.1371/journal.pone.0105992. 25171179 10.1371/journal.pone.0105992 PMC4149475

[105] Bosso L, Di Febbraro M, Cristinzio G, Zoina A, Russo D. Shedding light on the effects of climate change on the potential distribution of *Xylella fastidiosa* in the mediterranean basin. Biol Invasions. 2016;18(6):1759–68. 10.1007/s10530-016-1118-1.

[106] Smeraldo S, Di Febbraro M, Bosso L, Flaquer C, Guixé D, Lisón F, et al. Ignoring seasonal changes in the ecological niche of non-migratory species May lead to biases in potential distribution models: lessons from bats. Biodivers Conserv. 2018;27(9):2425–41. 10.1007/s10531-018-1545-7.

[107] Zhang J, Jiang F, Li G, Qin W, Li S, Gao H, et al. Maxent modeling for predicting the Spatial distribution of three raptors in the Sanjiangyuan National park, China. Ecol Evol. 2019;9(11):6643–54. 10.1002/ece3.5243. 31236249 10.1002/ece3.5243 PMC6580265

[108] Kuhn M. Building predictive models in R using the caret package. J Stat Softw. 2008;28:1–26.27774042

[109] Jinga P, Palagi J, Chong JP, Bobo ED. Climate change reduces the natural range of African wild Loquat (Uapaca Kirkiana müll. Arg., Phyllanthaceae) in south-central Africa. Reg Envriron Chang. 2020;20(3):108.

[110] Franklin J. Mapping species distributions: Spatial inference and prediction. Cambridge: Cambridge University Press; 2010.

[111] Morueta-Holme N, Fløjgaard C, Svenning JC. Climate change risks and conservation implications for a threatened small-range mammal species. PLoS ONE. 2010;5(4):e10360. 10.1371/journal.pone.0010360. 20454451 10.1371/journal.pone.0010360 PMC2861593

[112] De Marco P, Nóbrega CC. Evaluating collinearity effects on species distribution models: an approach based on virtual species simulation. PLoS ONE. 2018;13(9):e0202403. 10.1371/journal.pone.0202403. 30204749 10.1371/journal.pone.0202403 PMC6133275

[113] Ortega-Huerta MA, Peterson AT. Modeling ecological niches and predicting geographic distributions: A test of six presence-only methods. Rev Mex Biodivers. 2008;1(1):205–16. 10.22201/ib.20078706e.2008.1.17.

[114] Hernandez PA, Graham CH, Master LL, Albert DL. The effect of sample size and species characteristics on performance of different species distribution modeling methods. Ecography. 2006;29(5):773–85. 10.1111/j.0906-7590.2006.04700.x.

[115] Papeş M, Gaubert P. Modelling ecological niches from low numbers of occurrences: assessment of the conservation status of poorly known viverrids (Mammalia, Carnivora) across two continents. Divers Distrib. 2007;13(6):890–902. 10.1111/j.1472-4642.2007.00392.x.

[116] Valavi R, Guillera-Arroita G, Lahoz‐Monfort JJ, Elith J. Predictive performance of presence‐only species distribution models: a benchmark study with reproducible code. Ecol Monogr. 2022;92(1):e01486.

[117] Kaky E, Nolan V, Alatawi A, Gilbert F. A comparison between ensemble and maxent species distribution modelling approaches for conservation: A case study with Egyptian medicinal plants. Ecol Inf. 2020;60:101150.

[118] Yi Y, Cheng X, Yang ZF, Zhang SH. Maxent modeling for predicting the potential distribution of endangered medicinal plant (*H. riparia* Lour) in yunnan, China. Ecol Eng. 2016;92:260–9. 10.1016/j.ecoleng.2016.04.010.

[119] Fois M, Cuena-Lombraña A, Fenu G, Cogoni D, Bacchetta G. Does a correlation exist between environmental suitability models and plant population parameters? An experimental approach to measure the influence of disturbances and environmental changes. Ecol Ind. 2018;86:1–8. 10.1016/j.ecolind.2017.12.009.

[120] Allouche O, Tsoar A, Kadmon R. Assessing the accuracy of species distribution models: prevalence, kappa and the true skill statistic (TSS). J Appl Ecol. 2006;43:1223–32. 10.1111/j.1365-2664.2006.01214.x.

[121] Pearson RG, Raxworthy CJ, Nakamura M, Townsend Peterson AT. Predicting species distributions from small numbers of occurrence records: A test case using cryptic geckos in Madagascar. J Biogeogr. 2007;34(1):102–17. 10.1111/j.1365-2699.2006.01594.x.

[122] Choudhury MR, Deb P, Singha H, Chakdar B, Medhi M. Predicting the probable distribution and threat of invasive *Mimosa diplotricha* Suavalle and *Mikania micrantha* Kunth in a protected tropical grassland. Ecol Eng. 2016;97:23–31. 10.1016/j.ecoleng.2016.07.018.

[123] Xu W, Zhu S, Yang T, Cheng J, Jin J. Maximum entropy niche-based modeling for predicting the potential suitable habitats of a traditional medicinal plant (*Rheum nanum*) in Asia under climate change conditions. Agriculture. 2022;12(5):610. 10.3390/agriculture12050610.

[124] Kluge J, Worm S, Lange S, Long D, Böhner J, Yangzom R, et al. Elevational seed plant richness patterns in bhutan, Eastern himalaya. J Biogeogr. 2017;44(8):1711–22. 10.1111/jbi.12955.

[125] Hu Y, Jin K, Huang Z, Ding Z, Liang J, Pan X, et al. Elevational patterns of non-volant small mammal species richness in Gyirong valley, central himalaya: evaluating multiple Spatial and environmental drivers. J Biogeogr. 2017;44(12):2764–77. 10.1111/jbi.13102.

[126] Hu Y, Ding Z, Jiang Z, Quan Q, Guo K, Tian L, et al. Birds in the himalayas: what drives beta diversity patterns along an elevational gradient? Ecol Evol. 2018;8(23):11704–16. 10.1002/ece3.4622. 30598768 10.1002/ece3.4622 PMC6303779

[127] Zu K, Luo A, Shrestha N, Liu B, Wang Z, Zhu X. Altitudinal biodiversity patterns of seed plants along Gongga mountain in the southeastern Qinghai–Tibetan plateau. Ecol Evol. 2019;9(17):9586–96. 10.1002/ece3.5483. 31534677 10.1002/ece3.5483 PMC6745871

[128] Khedr A. Assessment, classification, and analysis of microhabitats supporting globally significant plant species. Conservation and sustainable use of medicinal plants in arid and semi-arid ecosystems project. Saint Katherine Protectorate, Egypt. Cairo, Egypt: Final Report: EEAA, GEF & UNDP; 2007.

[129] Qian H, Qian S, Zhang J, Kessler M. Effects of climate and environmental heterogeneity on the phylogenetic structure of regional angiosperm floras worldwide. Nat Commun. 2024;15(1):1079. 10.1038/s41467-024-45155-9. 38316752 10.1038/s41467-024-45155-9 PMC10844608

[130] Gebrehiwot K, Demissew S, Woldu Z, Fekadu M, Desalegn T, Teferi E. Elevational changes in vascular plants richness, diversity, and distribution pattern in abune Yosef mountain range, Northern Ethiopia. Plant Divers. 2019;41(4):220–8. 10.1016/j.pld.2019.06.005. 31528781 10.1016/j.pld.2019.06.005 PMC6743012

[131] de Lima RAF, Souza VC, de Siqueira MF, ter Steege H. Defining endemism levels for biodiversity conservation: tree species in the Atlantic forest hotspot. Biol Conserv. 2020;252:108825.

[132] Werneck M, de Sobral S, Rocha MEG, Landau CTV, Stehmann EC, J.R. Distribution and endemism of angiosperms in the Atlantic forest. Nat Conserv. 2011;9:188–93.

[133] Abdelaal M, Fois M, Fenu G, Bacchetta G. Critical checklist of the endemic vascular plants of Egypt. Phytotaxa. 2018;360(1):19–34. 10.1164/phytotaxa.360.1.2.

[134] El-Khalafy MM, Shaltout KH, Ahmed DA. Updating and assessing plant endemism in Egypt. Phytotaxa. 2021;502(3):237–58.

[135] Abd El-Ghani M, Hosni H, Shamso E, Ellmouni F. New perspectives, additions, and amendments to plant endemism in a North African flora. Bot Stud. 2024;65(1):21. 39012376 10.1186/s40529-024-00428-w PMC11252113

[136] Moradi H, Noroozi J, Fourcade Y. Plant endemic diversity in the Irano-Anatolian global biodiversity hotspot is dramatically threatened by future climate change. Biol Conserv. 2025;302:110963.

[137] Meng HH, Zhou SS, Jiang XL, Gugger PF, Li L, Tan YH, Li J. Are mountaintops climate refugia for plants under global warming? A lesson from high-mountain Oaks in tropical rainforest. Alp Bot. 2019;129:175–83.

[138] Hughes L. Climate change and australia: trends, projections and impacts. Austral Ecol. 2003;28(4):423–43. 10.1046/j.1442-9993.2003.01300.x.

[139] Luo Z, Jiang Z, Tang S. Impacts of climate change on distributions and diversity of ungulates on the Tibetan plateau. Ecol Appl. 2015;25(1):24–38. 10.1890/13-1499.1. 26255355 10.1890/13-1499.1

[140] Manish K, Telwala Y, Nautiyal DC, Pandit MK. Modelling the impacts of future climate change on plant communities in the himalaya: a case study from Eastern himalaya, India. Model Earth Syst Environ. 2016;2(92):1–12. 10.1007/s40808-016-0163-1.

[141] Di Musciano M, Zannini P, Ferrara C, Spina L, Nascimbene J, Vetaas OR, et al. Investigating elevational gradients of species richness in a mediterranean plant hotspot using a published flora. Front Biogeogr. 2021;13(3). 10.21425/F5FBG50007.

[142] Pearson RG, Dawson TP. Predicting the impacts of climate change on the distribution of species: are bioclimate envelope models useful? Glob Ecol Biogeogr. 2003;12(5):361–71. 10.1046/j.1466-822X.2003.00042.x.

[143] Vasconcelos TS, Rodríguez MA, Hawkins BA. Species distribution modelling as a macroecological tool: a case study using new world amphibians. Ecography. 2012;35(6):539–48. 10.2307/41510693.

