# Supplementary material for: Future habitat dynamics of critically endangered endemic plants in the St. Catherine protected area, South Sinai, Egypt: climate change perspectives on mountain ecosystems
Source: BMC Ecol Evol. 2025 Jul 10;25:68. doi: 10.1186/s12862-025-02408-5 (PMC12243221; doi:10.1186/s12862-025-02408-5)
Supplement: Supplementary file 1 — Supplementary Material 1. [file 12862_2025_2408_MOESM1_ESM.docx]

Supplementary materials

**Tables**

Table S1: Minimum, Maximum, and standard deviation (SD) of the soil characteristics target species in St. Catherine Protected Area.

|  | *P. boveana* | | | *R. arabica* | | | *M. serbaliana* | | | *S. oreosinaica* | | |
| --- | --- | --- | --- | --- | --- | --- | --- | --- | --- | --- | --- | --- |
| Variable | Min. | Max. | S.D | Min. | Max. | S.D | Min. | Max. | S.D | Max. | Min. | S.D |
| Water content % | 0.94 | 4.8 | 1.33 | 0.31 | 1.6 | 0.34 | 0.31 | 1.58 | 0.3 | 32 | 0.08 | 3.6 |
| pH | 7.6 | 8.9 | 0.48 | 7.4 | 8.9 | 0.42 | 7.1 | 8.8 | 0.3 | 8.9 | 7.1 | 0.4 |
| T.D.S PPm | 80 | 250 | 59.65 | 18.27 | 365 | 72.67 | 38 | 1400 | 247.1 | 1730 | 34.62 | 221.3 |
| EC µs/ cm | 104 | 550 | 175.78 | 38 | 649 | 142.29 | 18.27 | 678 | 146.5 | 2940 | 72 | 399.9 |
| Org. matter % | 1.76 | 8.03 | 2.04 | 0.23 | 17.25 | 3.62 | 0.45 | 17.25 | 3.5 | 17.08 | 0.23 | 3.9 |
| CaCO_3_% | 12.5 | 36.33 | 10.22 | 10 | 47 | 8.62 | 7.5 | 47 | 9.9 | 47 | 2.5 | 9 |
| Ca^++^meq/L | 1.9 | 53 | 18.2 | 1.5 | 53 | 12.58 | 1.5 | 50 | 12.3 | 50 | 1 | 11 |
| Mg^++^ meq/L | 1 | 11 | 3.66 | 0.5 | 52.5 | 9.93 | 0.5 | 52.5 | 10.3 | 52.5 | 0.3 | 8.5 |
| Na^+^ PPM | 10.4 | 45 | 13.48 | 10.4 | 49.5 | 10.49 | 10.4 | 53.64 | 11.3 | 65 | 9.2 | 11.9 |
| K^+^PPM | 10.4 | 50 | 13.12 | 10.4 | 57.9 | 12.13 | 10.4 | 53.64 | 11.6 | 80.01 | 9.11 | 14.8 |
| HCO3^-^ meq/L | 4 | 13 | 3 | 4 | 16 | 2.89 | 4 | 20 | 4.5 | 20 | 4 | 3.6 |
| Cl-meq/L | 2.75 | 14.5 | 4.32 | 2.75 | 18.5 | 3.72 | 2.25 | 15.5 | 4.0 | 52.25 | 2.25 | 6.9 |
| SO_4_^--^ meq/l | 16.5 | 133 | 42.48 | 5 | 160 | 32.98 | 3 | 160 | 33.9 | 160 | 3 | 30.3 |

Table S2. Threats on the target species based on IUCN Threats Classification Scheme

| Code | Threat | Timing | Scope | Severity | Score |
| --- | --- | --- | --- | --- | --- |
| *Primula boveana* | | | | | |
| 1.3 | Residential & commercial development -> Tourism & recreation areas | Ongoing | Minority (<50%) | Causing/Could cause fluctuations | Moderate Impact: 7 |
| 5.2.3 | Gathering terrestrial plants -> Persecution/control | Ongoing | Minority (<50%) | Causing/Could cause fluctuations | Low Impact: 5 |
| 6.3 | Human intrusions & disturbance -> Work & other activities | Ongoing | Whole (<90%) | Very Rapid Declines | High Impact: 9 |
| 7.2.5. | Natural system modifications -> Dams & water management/use -> Abstraction of ground water (domestic use) | Ongoing | Whole (<90%) | Slow, Significant Declines | High Impact: 9 |
| 11.1 | Climate change & severe weather -> Habitat shifting & alteration | Ongoing | Whole (>90%) | Causing/Could cause fluctuations | Low Impact: 5 |
| 11.2 | Climate change & severe weather -> Droughts | Ongoing | Whole (>90%) | Very Rapid Declines | High Impact: 9 |
| 11.3 | Climate change & severe weather -> Temperature extremes | Ongoing | Whole (<90%) | Very Rapid Declines | High Impact: 9 |
| 11.4 | Climate change & severe weather -> Storms & flooding | Ongoing | Whole (>90%) | Very Rapid Declines | High Impact: 9 |
| *Rosa arabica* | | | | | |
| 1.3 | Residential & commercial development -> Tourism & recreation areas | Ongoing | Minority (<50%) | Causing/Could cause fluctuations | Moderate Impact: 7 |
| 2.3.1 | Agriculture & aquaculture -> Livestock farming & ranching -> Nomadic grazing | Ongoing | Minority (<50%) | Causing/Could cause fluctuations | Moderate Impact: 7 |
| 5.2.3 | Gathering terrestrial plants -> Persecution/control | Ongoing | Minority (<50%) | Causing/Could cause fluctuations | Low Impact: 5 |
| 6.3 | Human intrusions & disturbance -> Work & other activities | Ongoing | Whole (<90%) | Very Rapid Declines | High Impact: 9 |
| 7.2.5. | Natural system modifications -> Dams & water management/use -> Abstraction of ground water (domestic use) | Ongoing | Whole (<90%) | Slow, Significant Declines | High Impact: 9 |
| 11.2. | Climate change & severe weather -> Droughts | Ongoing | Whole (>90%) | Very Rapid Declines | High Impact: 9 |
| 11.3 | Climate change & severe weather -> Temperature extremes | Ongoing | Whole (<90%) | Very Rapid Declines | High Impact: 9 |
| 11.4 | Climate change & severe weather -> Storms & flooding | Ongoing | Whole (>90%) | Very Rapid Declines | High Impact: 9 |
| *Micromeria* and *Silene* | | | | | |
| 2.3.1 | Agriculture & aquaculture -> Livestock farming & ranching -> Nomadic grazing | Ongoing | Minority (<50%) | Causing/Could cause fluctuations | Moderate Impact: 7 |
| 6.1. | Human intrusions & disturbance -> Recreational activities | Ongoing | Minority (<50%) | Causing/Could cause fluctuations | Low Impact: 5 |
| 7.2.5. | Natural system modifications -> Dams & water management/use -> Abstraction of ground water (domestic use) | Ongoing | Whole (<90%) | Slow, Significant Declines | High Impact: 9 |
| 11.2. | Climate change & severe weather -> Droughts | Ongoing | Whole (>90%) | Very Rapid Declines | High Impact: 9 |

Table S3. Model performance for the target species

|  | Time |  | Scenario | Average | Std. Deviation |
| --- | --- | --- | --- | --- | --- |
| ***Primula boveana*** | | | | | |
| AUC (Training) | |  | Current | 0.97 | 0.00 |
|  | 2050 |  | ssp12.6 | 0.97 | 0.00 |
|  |  |  | ssp58.5 | 0.97 | 0.00 |
|  | 2070 |  | ssp12.6 | 0.97 | 0.00 |
|  |  |  | ssp58.5 | 0.97 | 0.00 |
| AUC (Test) | |  | Current | 0.96 | 0.05 |
|  | 2050 |  | ssp12.6 | 0.96 | 0.06 |
|  |  |  | ssp58.5 | 0.96 | 0.05 |
|  | 2070 |  | ssp12.6 | 0.96 | 0.05 |
|  |  |  | ssp58.5 | 0.96 | 0.05 |
| TSS | |  | Current | 0.92 | 0.04 |
| Threshold 0.312 | 2050 |  | ssp12.6 | 0.92 | 0.05 |
|  |  |  | ssp58.5 | 0.91 | 0.07 |
|  | 2070 |  | ssp12.6 | 0.92 | 0.05 |
|  |  |  | ssp58.5 | 0.91 | 0.04 |
| ***Rosa arabica*** | | | | | |
| AUC (Training) | |  | Current | 0.98 | 0.00 |
|  | 2050 |  | ssp12.6 | 0.98 | 0.00 |
|  |  |  | ssp58.5 | 0.98 | 0.00 |
|  | 2070 |  | ssp12.6 | 0.98 | 0.00 |
|  |  |  | ssp58.5 | 0.98 | 0.00 |
| AUC (Test) | |  | Current | 0.96 | 0.04 |
|  | 2050 |  | ssp12.6 | 0.96 | 0.04 |
|  |  |  | ssp58.5 | 0.96 | 0.05 |
|  | 2070 |  | ssp12.6 | 0.96 | 0.05 |
|  |  |  | ssp58.5 | 0.96 | 0.04 |
| TSS | |  | Current | 0.91 | 0.11 |
| Threshold 0.482 | 2050 |  | ssp12.6 | 0.90 | 0.11 |
|  |  |  | ssp58.5 | 0.90 | 0.12 |
|  | 2070 |  | ssp12.6 | 0.91 | 0.11 |
|  |  |  | ssp58.5 | 0.91 | 0.12 |
| ***Micromeria serbaliana*** | | | | | |
| AUC (Training) | |  | Current | 0.98 | 0.00 |
|  | 2050 |  | ssp12.6 | 0.98 | 0.00 |
|  |  |  | ssp58.5 | 0.98 | 0.00 |
|  | 2070 |  | ssp12.6 | 0.98 | 0.00 |
|  |  |  | ssp58.5 | 0.98 | 0.00 |
| AUC (Test) | |  | Current | 0.97 | 0.03 |
|  | 2050 |  | ssp12.6 | 0.97 | 0.03 |
|  |  |  | ssp58.5 | 0.97 | 0.03 |
|  | 2070 |  | ssp12.6 | 0.97 | 0.04 |
|  |  |  | ssp58.5 | 0.97 | 0.03 |
| TSS | |  | Current | 0.89 | 0.19 |
| Threshold 0.40 | 2050 |  | ssp12.6 | 0.89 | 0.21 |
|  |  |  | ssp58.5 | 0.88 | 0.20 |
|  | 2070 |  | ssp12.6 | 0.89 | 0.20 |
|  |  |  | ssp58.5 | 0.88 | 0.19 |
| ***Silene oreosinaica*** | | | | | |
| AUC (Training) | |  | Current | 0.97 | 0.00 |
|  | 2050 |  | ssp12.6 | 0.96 | 0.01 |
|  |  |  | ssp58.5 | 0.97 | 0.01 |
|  | 2070 |  | ssp12.6 | 0.97 | 0.01 |
|  |  |  | ssp58.5 | 0.96 | 0.01 |
| AUC (Test) | |  | Current | 0.96 | 0.06 |
|  | 2050 |  | ssp12.6 | 0.95 | 0.06 |
|  |  |  | ssp58.5 | 0.96 | 0.06 |
|  | 2070 |  | ssp12.6 | 0.95 | 0.06 |
|  |  |  | ssp58.5 | 0.95 | 0.08 |
| TSS | |  | Current | 0.85 | 0.21 |
| Threshold 0.30 | 2050 |  | ssp12.6 | 0.86 | 0.22 |
|  |  |  | ssp58.5 | 0.85 | 0.21 |
|  | 2070 |  | ssp12.6 | 0.85 | 0.23 |
|  |  |  | ssp58.5 | 0.85 | 0.23 |

Table S4. Environmental variables range for targets species under current climatic conditions

|  | ***Primula boveana*** | | | ***Rosa arabica*** | | | ***Micromeria serbaliana*** | | | ***Silene oreosinaica*** | | |
| --- | --- | --- | --- | --- | --- | --- | --- | --- | --- | --- | --- | --- |
| Variables | Min | Max | Average | Min | Max | Average | Min | Max | Average | Min | Max | Average |
| Bio1 | 12.53 | 15.69 | 14.06 | 13.57 | 16.10 | 15.04 | 13.85 | 15.92 | 14.58 | 12.53 | 19.56 | 14.19 |
| Bio2 | 11.71 | 12.76 | 12.37 | 11.43 | 12.32 | 11.80 | 11.52 | 12.39 | 11.93 | 11.25 | 12.52 | 12.24 |
| Bio3 | 41.46 | 43.54 | 42.53 | 40.80 | 42.33 | 41.51 | 40.87 | 42.58 | 41.67 | 41.51 | 42.60 | 42.34 |
| Bio4 | 650.51 | 666.78 | 659.07 | 649.71 | 662.26 | 654.46 | 650.46 | 661.58 | 657.39 | 610.86 | 666.78 | 655.80 |
| Bio5 | 26.90 | 30.10 | 28.32 | 27.80 | 29.80 | 28.93 | 27.90 | 29.80 | 28.55 | 26.90 | 32.90 | 28.34 |
| Bio6 | -2.50 | 1.20 | -0.80 | -1.30 | 1.80 | 0.50 | -1.00 | 1.40 | -0.08 | -2.50 | 5.80 | -0.56 |
| Bio7 | 28.20 | 29.40 | 29.09 | 28.00 | 29.10 | 28.43 | 28.20 | 29.10 | 28.64 | 27.10 | 29.40 | 28.90 |
| Bio8 | 4.87 | 11.30 | 6.79 | 5.95 | 11.80 | 8.61 | 6.27 | 11.63 | 7.29 | 4.87 | 13.10 | 6.73 |
| Bio9 | 19.70 | 22.70 | 21.32 | 20.98 | 23.37 | 22.26 | 21.23 | 23.20 | 21.92 | 19.70 | 25.35 | 21.29 |
| Bio10 | 19.98 | 22.98 | 21.44 | 20.98 | 23.37 | 22.36 | 21.23 | 23.20 | 21.92 | 19.98 | 26.45 | 21.53 |
| Bio11 | 3.93 | 7.28 | 5.55 | 5.02 | 7.68 | 6.58 | 5.32 | 7.50 | 6.08 | 3.93 | 11.65 | 5.72 |
| Bio12 | 53.00 | 105.00 | 78.29 | 50.00 | 85.00 | 64.81 | 50.00 | 81.00 | 70.08 | 16.00 | 105.00 | 80.00 |
| Bio13 | 10.00 | 19.00 | 14.67 | 10.00 | 16.00 | 12.22 | 10.00 | 15.00 | 13.22 | 3.00 | 19.00 | 14.79 |
| Bio14 | 0.00 | 1.00 | 0.86 | 0.00 | 1.00 | 0.59 | 0.00 | 1.00 | 0.92 | 0.00 | 1.00 | 0.86 |
| Bio15 | 64.69 | 68.42 | 66.61 | 63.70 | 70.91 | 66.66 | 64.35 | 70.91 | 67.40 | 55.83 | 68.26 | 65.43 |
| Bio16 | 25.00 | 49.00 | 37.14 | 25.00 | 41.00 | 31.13 | 25.00 | 38.00 | 33.64 | 8.00 | 49.00 | 37.93 |
| Bio17 | 2.00 | 6.00 | 3.95 | 1.00 | 4.00 | 2.72 | 1.00 | 4.00 | 3.19 | 0.00 | 6.00 | 4.00 |
| Bio18 | 3.00 | 7.00 | 4.38 | 1.00 | 4.00 | 3.06 | 1.00 | 4.00 | 3.19 | 0.00 | 7.00 | 4.29 |
| Bio19 | 22.00 | 43.00 | 32.33 | 21.00 | 36.00 | 27.19 | 21.00 | 33.00 | 29.44 | 7.00 | 43.00 | 33.21 |
| Elevation | 1861 | 2494 | 2182 | 1777 | 2279 | 1978 | 1804 | 2225 | 2068 | 883 | 2494 | 2129 |
| Slope | 5.92 | 12.97 | 9.94 | 1.76 | 12.97 | 6.23 | 1.18 | 14.97 | 8.76 | 4.76 | 12.43 | 8.90 |
| Aspect | 27.81 | 353.13 | 109.29 | 6.57 | 353.13 | 208.12 | 1.63 | 356.20 | 110.04 | 27.81 | 344.84 | 245.62 |
| Bulk | 135.22 | 138.02 | 135.90 | 134.27 | 138.02 | 135.64 | 135.18 | 136.83 | 136.16 | 135.22 | 136.31 | 135.47 |
| Clay | 264.85 | 350.93 | 294.02 | 279.26 | 365.33 | 320.20 | 282.62 | 380.73 | 317.13 | 264.85 | 323.75 | 282.22 |
| O.C. | 15.96 | 20.48 | 19.52 | 15.67 | 19.75 | 17.89 | 16.12 | 20.03 | 18.70 | 13.73 | 20.03 | 18.92 |
| Ph | 7.81 | 7.90 | 7.88 | 7.81 | 7.90 | 7.86 | 7.80 | 7.89 | 7.86 | 7.80 | 7.90 | 7.88 |

**Figures**

**
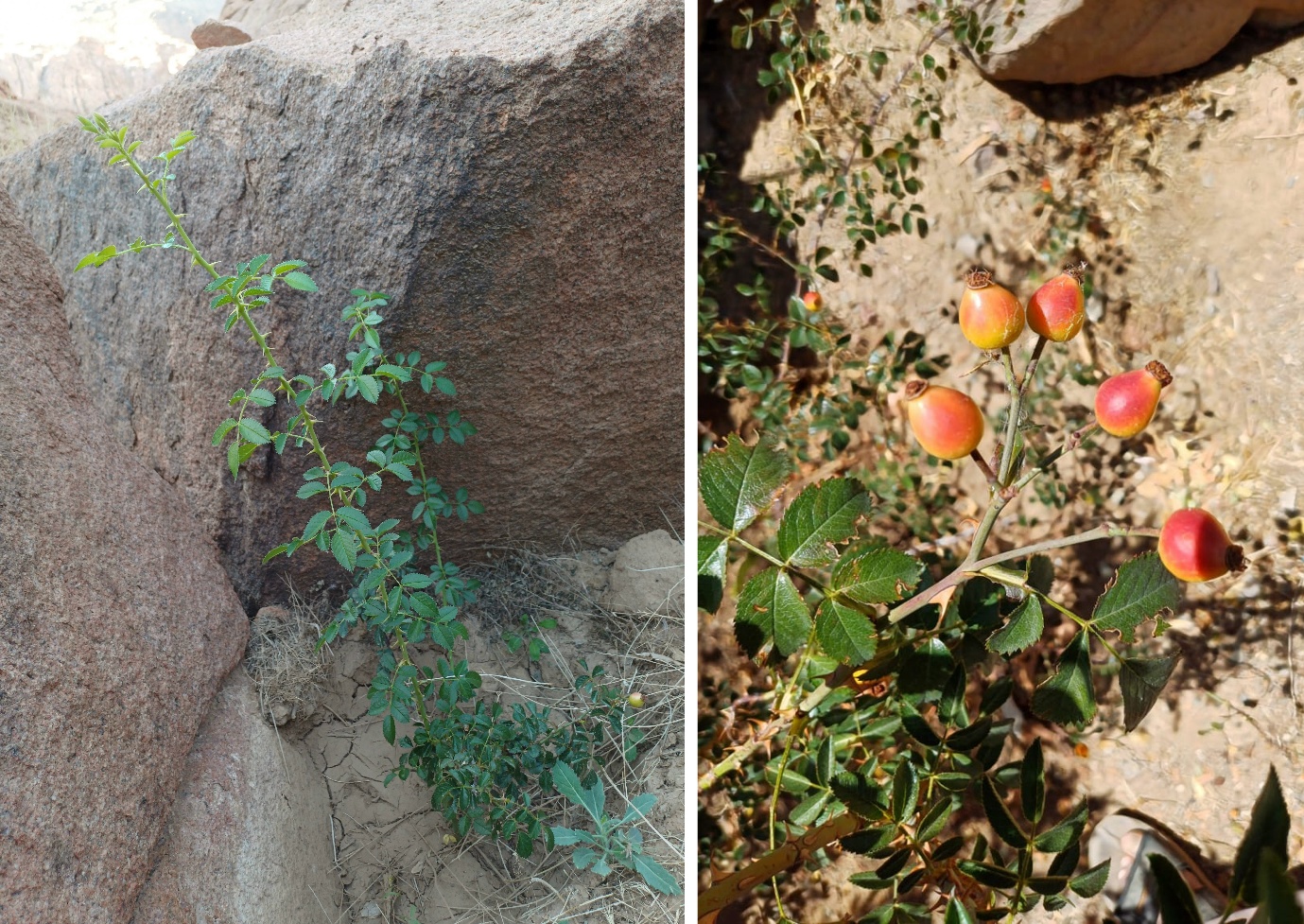
**

**
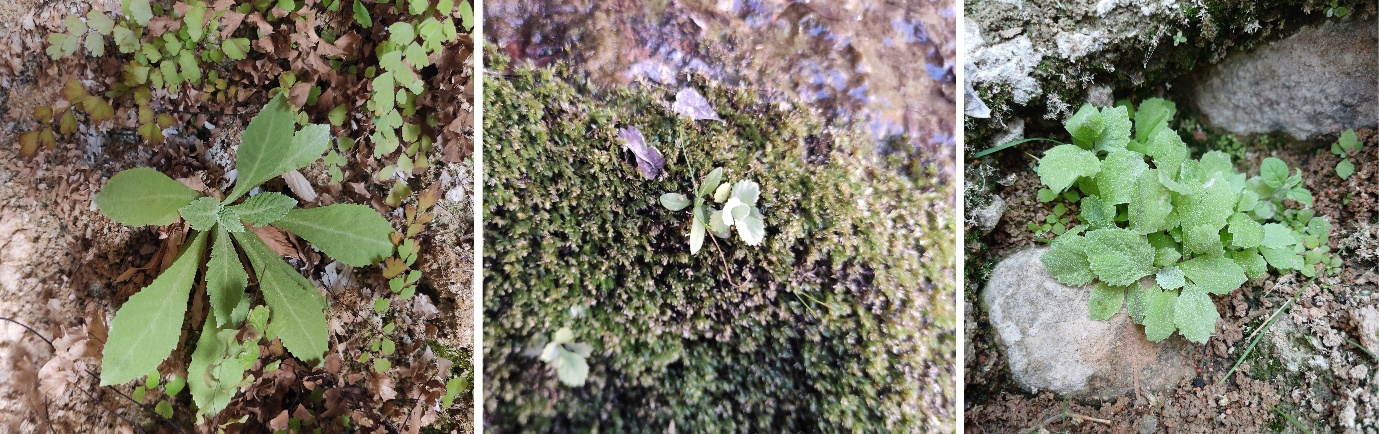
**

Fig. S.1. New individuals recently observed outside the historical geographical range of *R. arabica* and *P. boveana*

Fig. S.2. Observed reduction in number of individuals for *P. boveana*


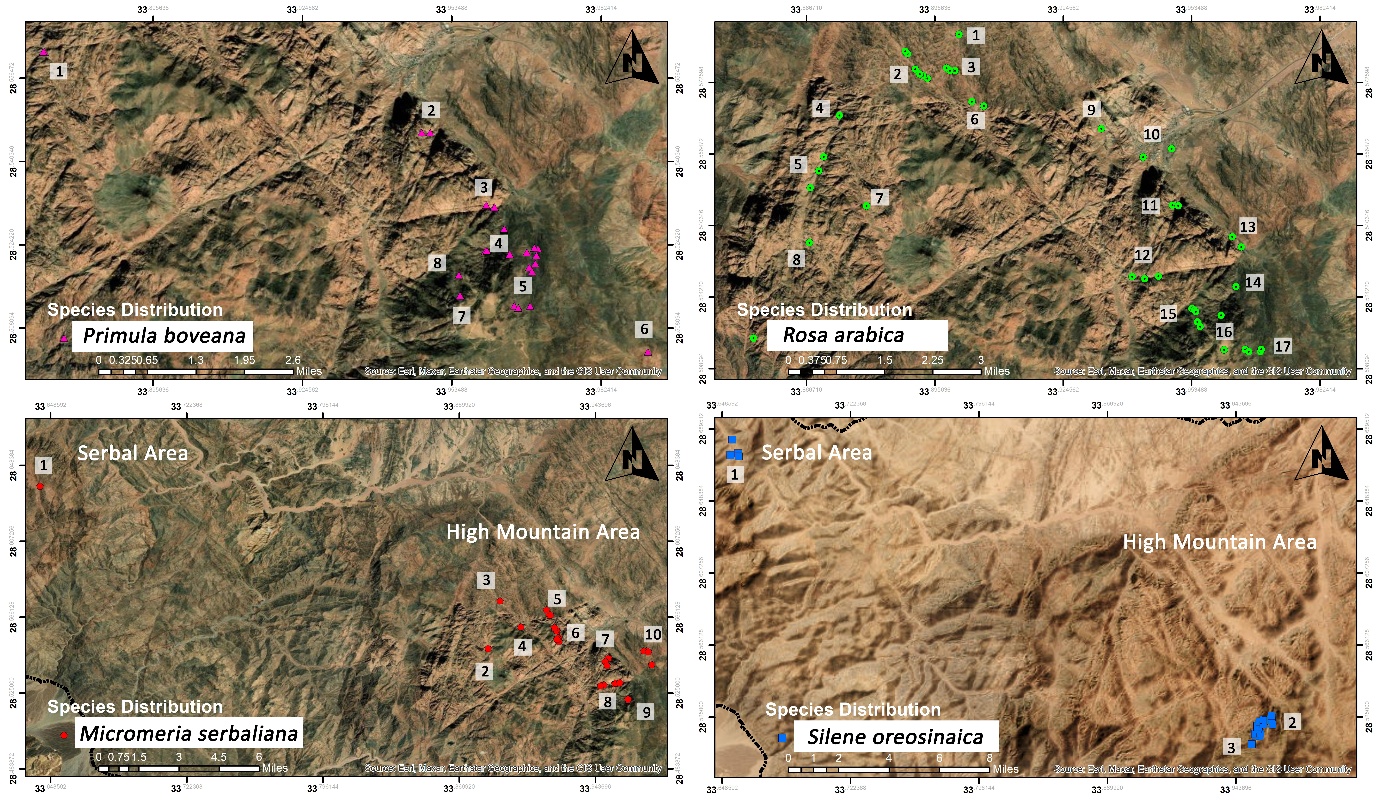


Fig. S.3. Main sub-populations for the target species

Fig. S.4. Microhabitat of the target endemic species (St. Catherine PA)

Fig. S.5. Impact of grazing on target endemic species


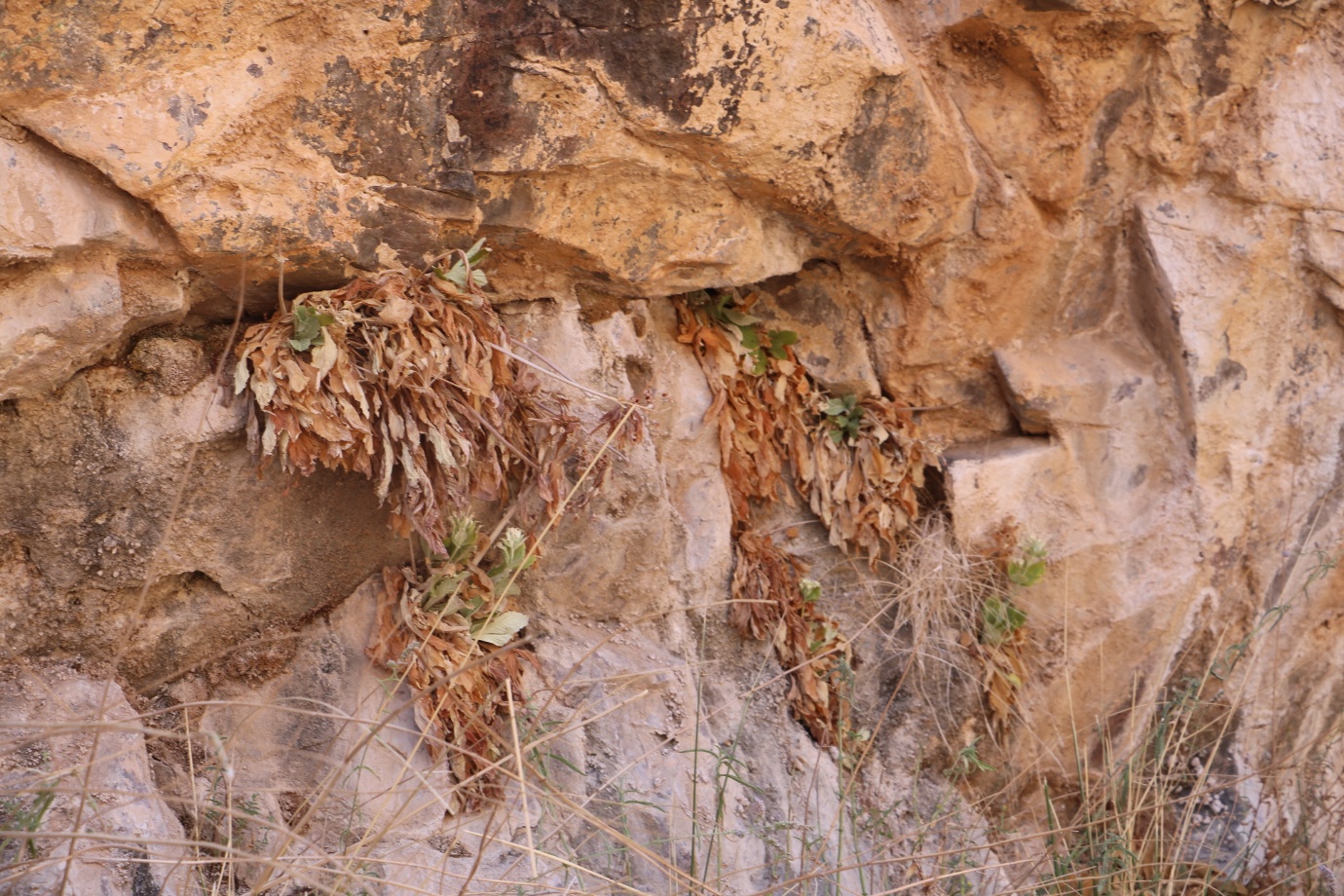


Fig. S.6. Drought impact on *P. boveana*

**
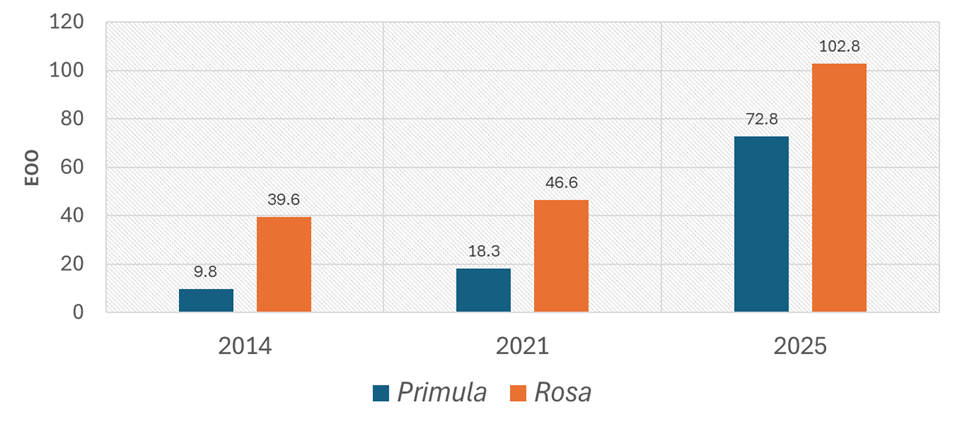
**

Fig. S.7. Trend of Extent of Occurrence (EOO) for *P. boveana* and *R. arabica*


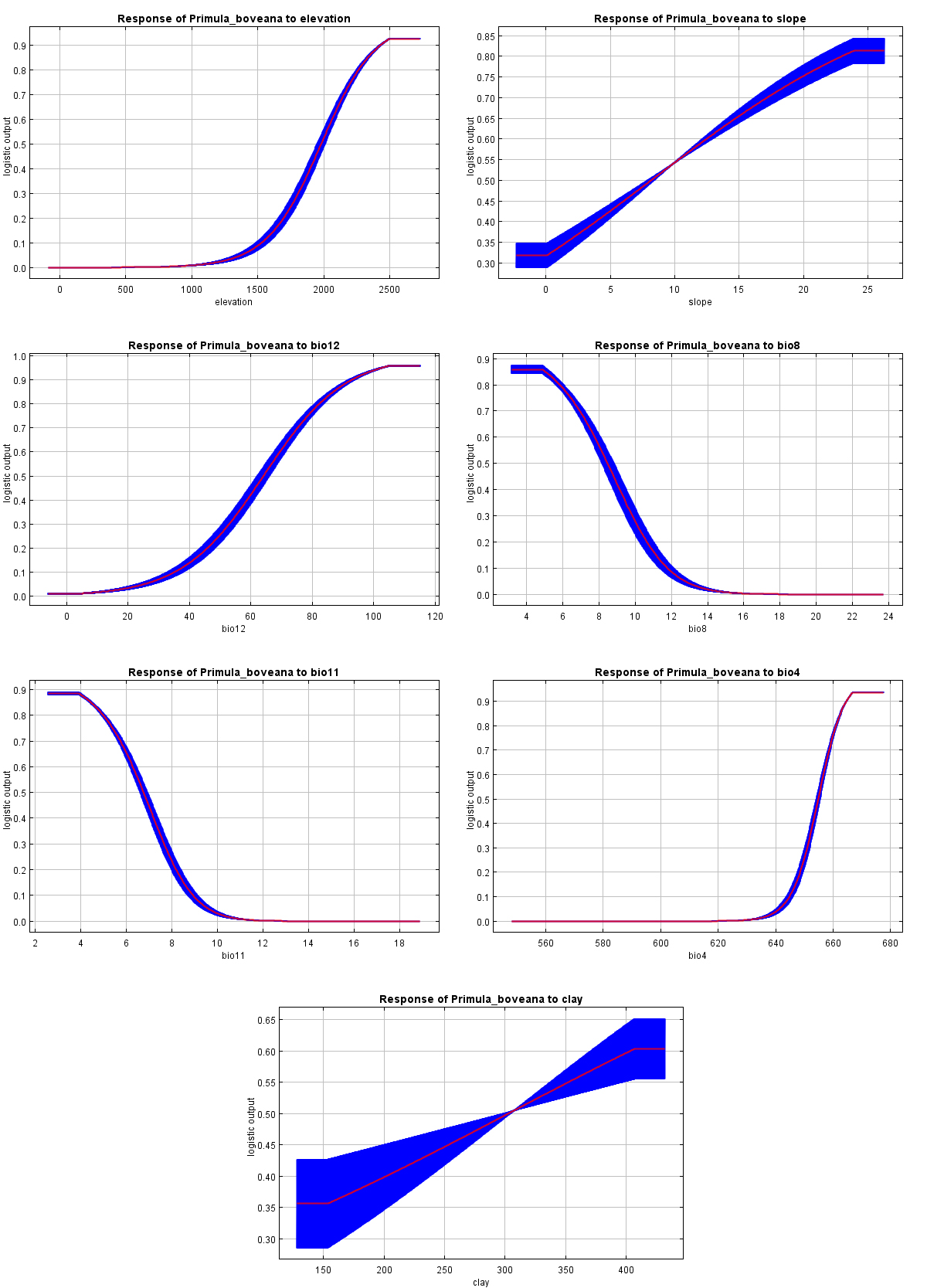


Fig. S.8. Response curves for the main environmental factors’ affection the distribution of *P. boveana*


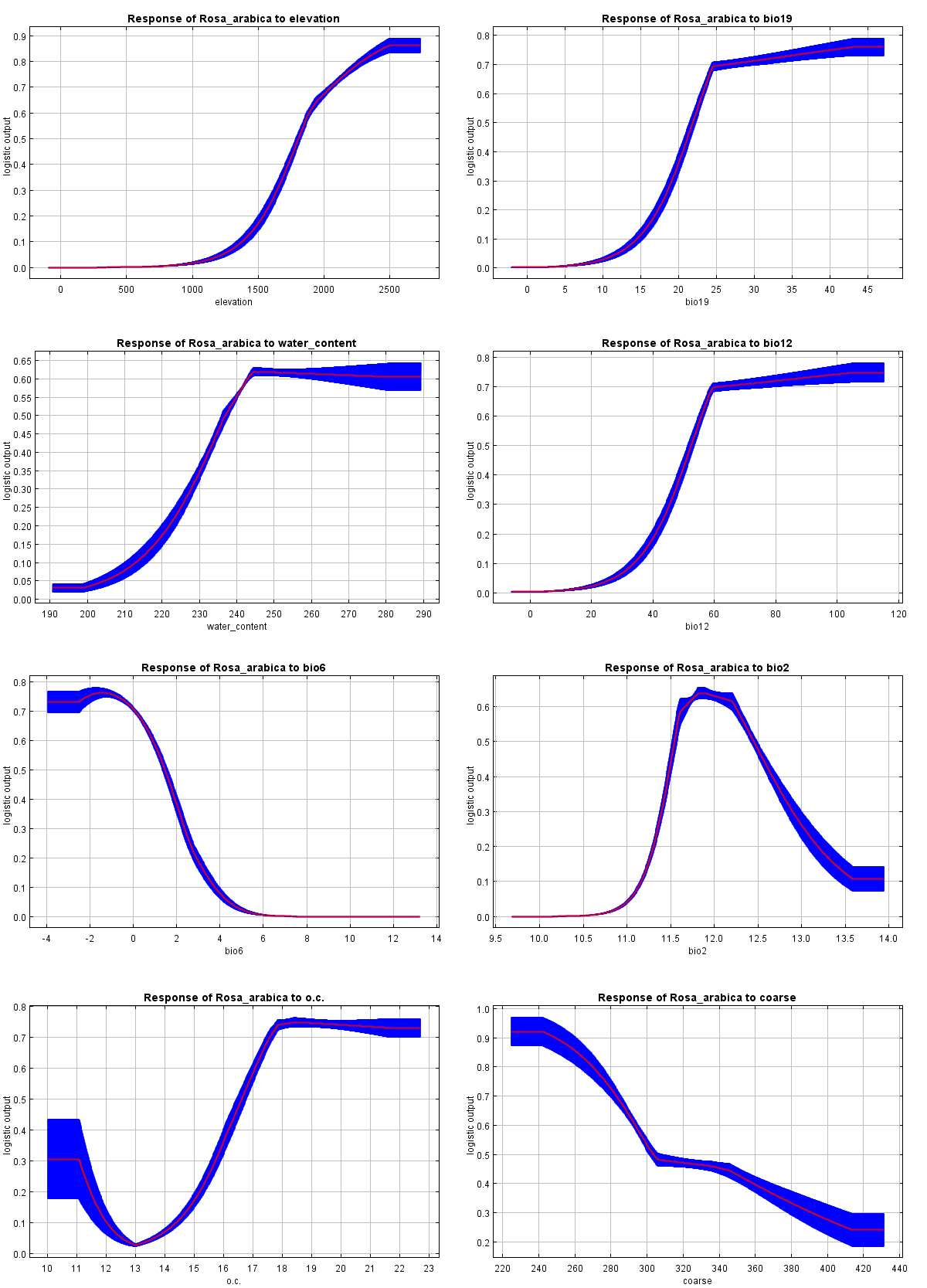


Fig. S.9. Response curves for the main environmental factors’ affection the distribution of *R. arabica*


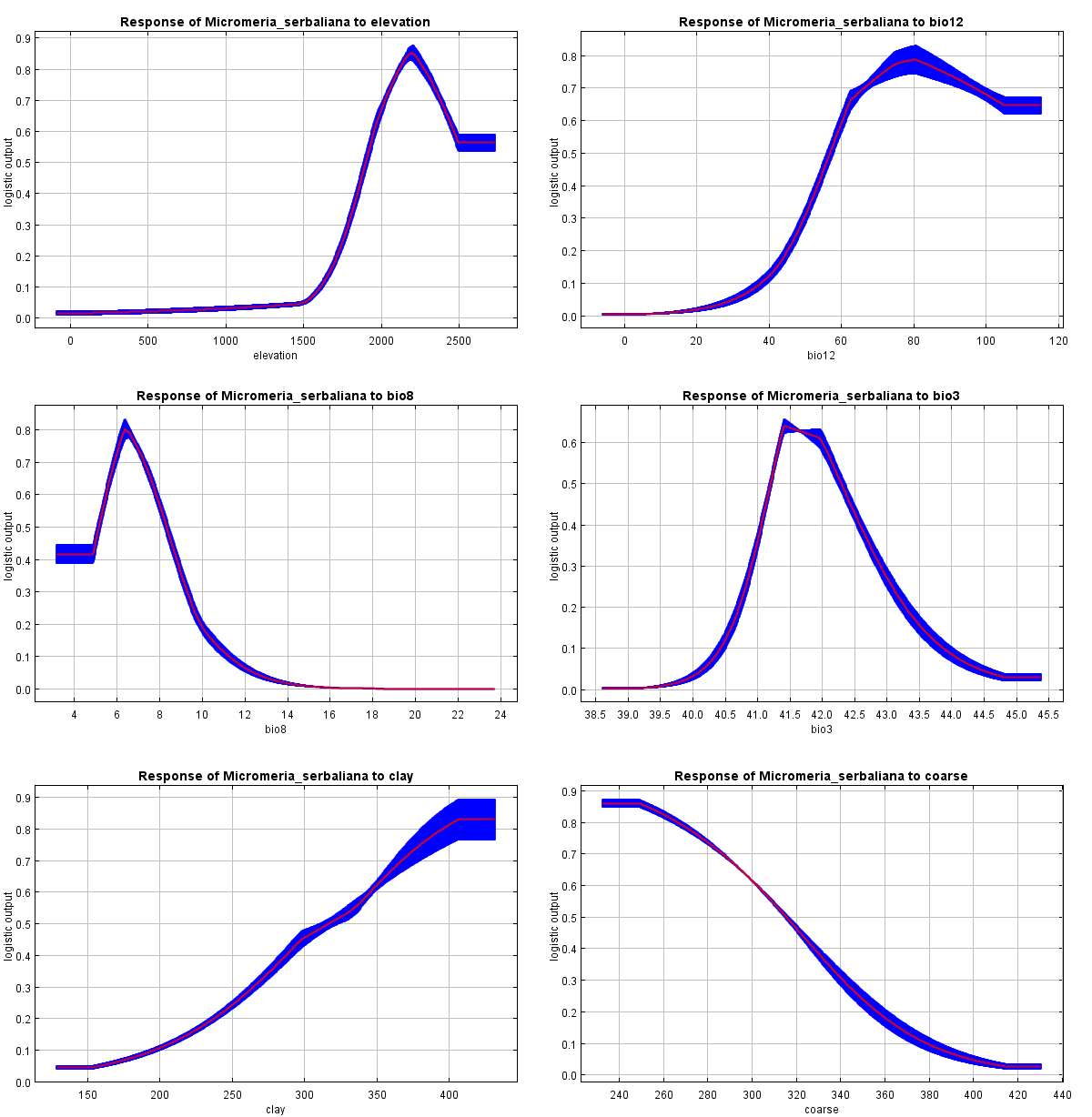


Fig. S.10. Response curves for the main environmental factors’ affection the distribution of *M. serbaliana*


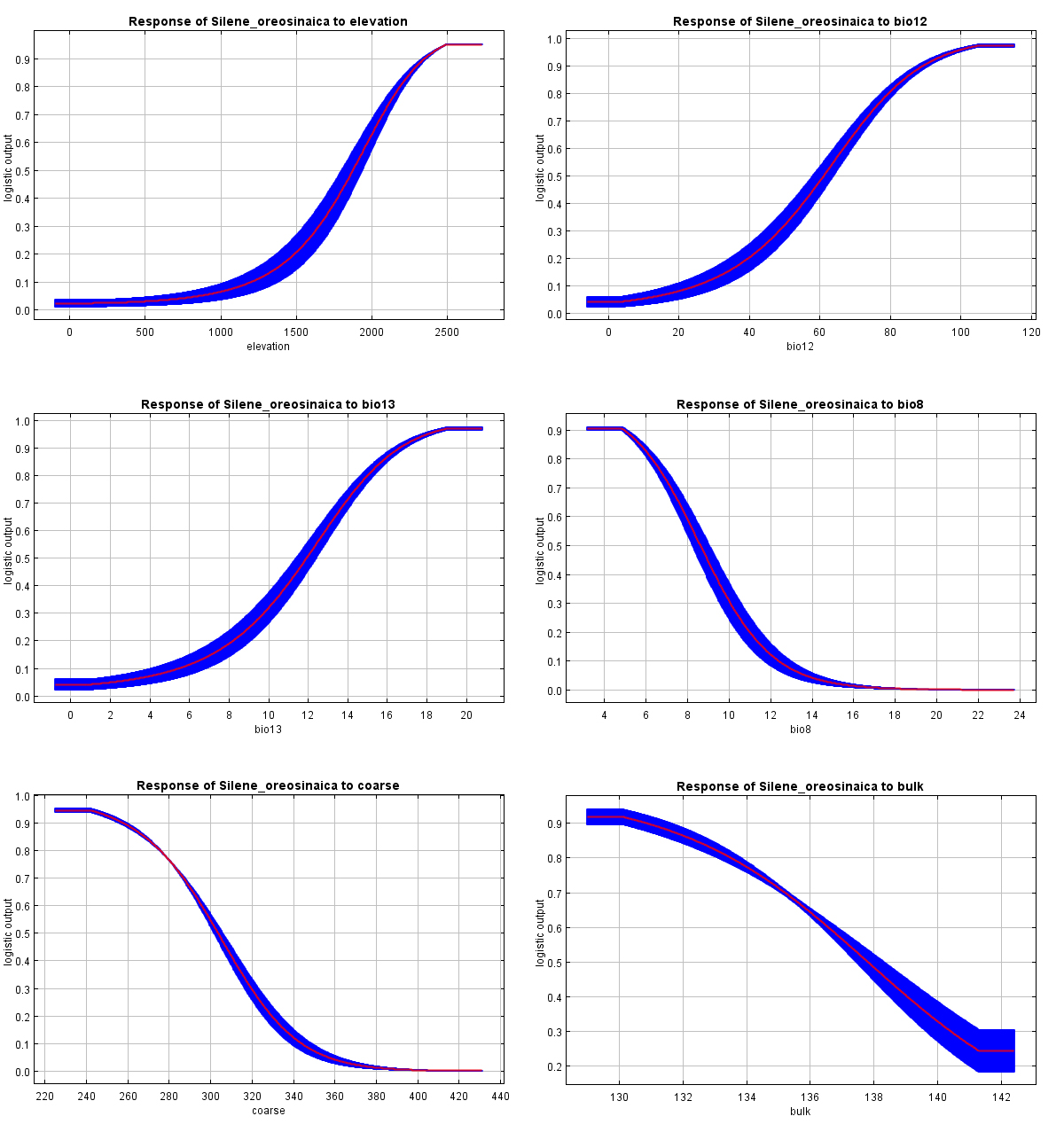


Fig. S11. Response curves for the main environmental factors’ affection the distribution of *Silene oreosinaica*


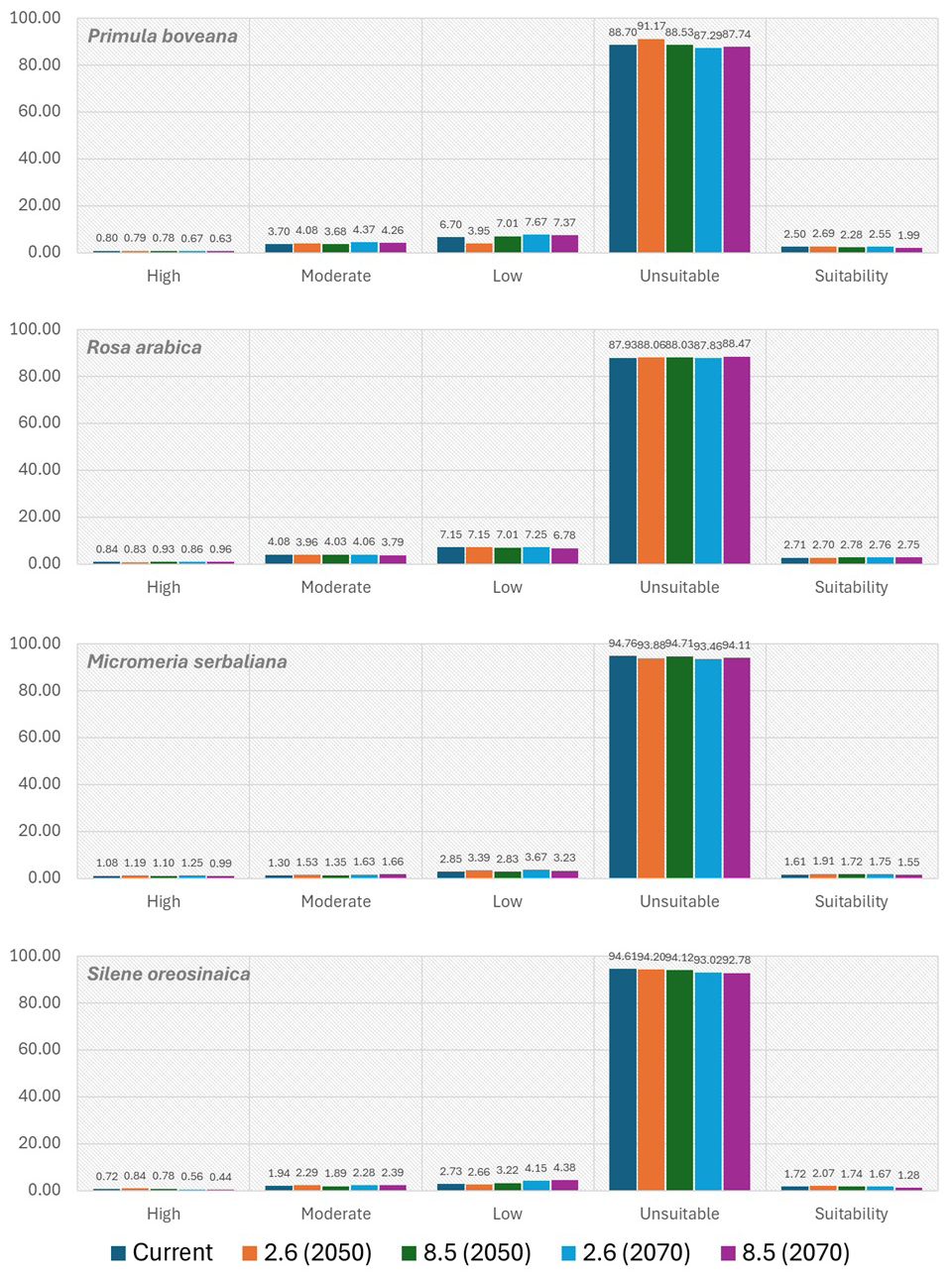


Fig. S12. Percentage of total changes in the probability areas for target species between current and future climatic scenarios


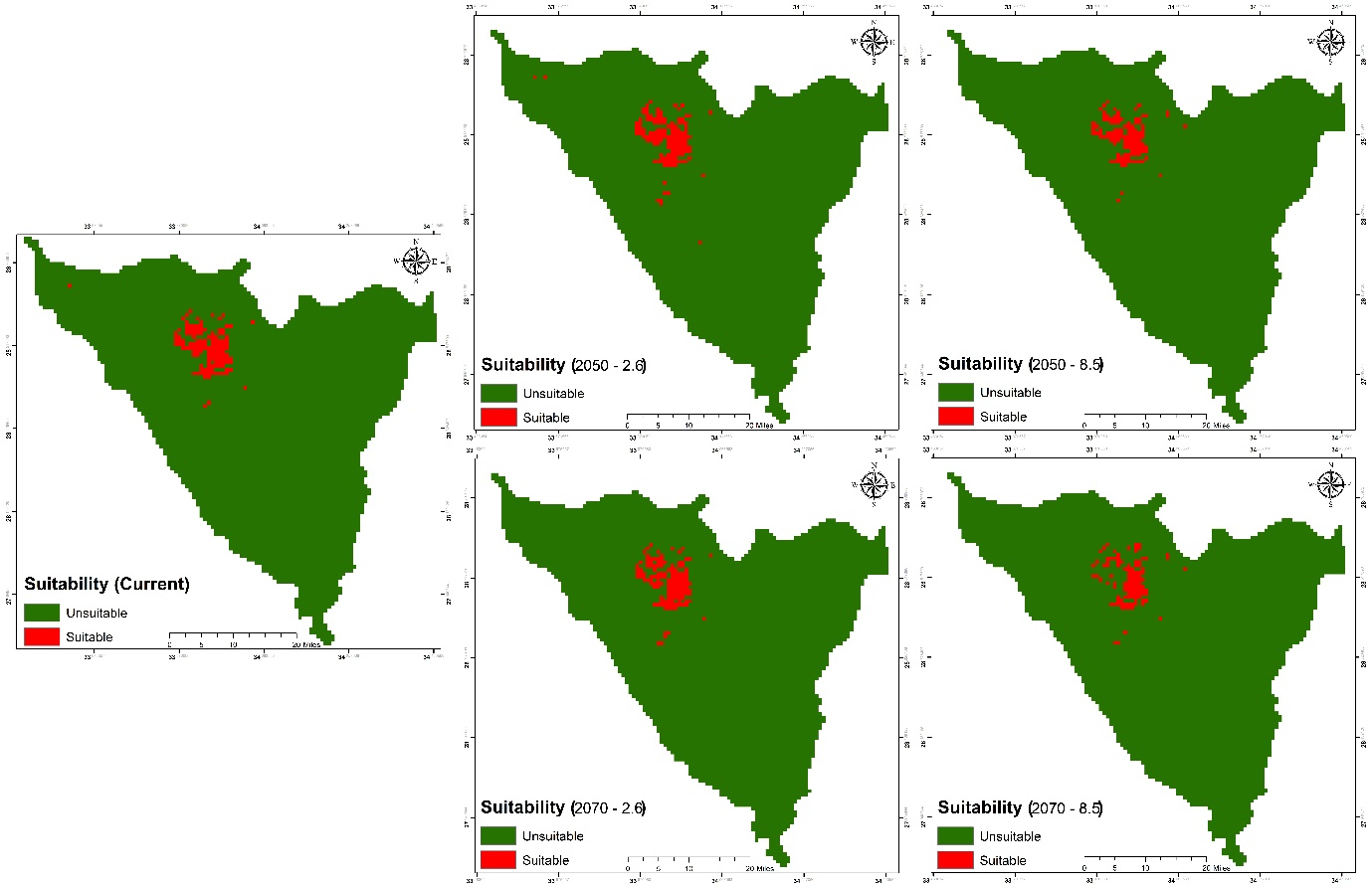


Fig. S13. Map of the current and future potential habitat for *Primula boveana*

*
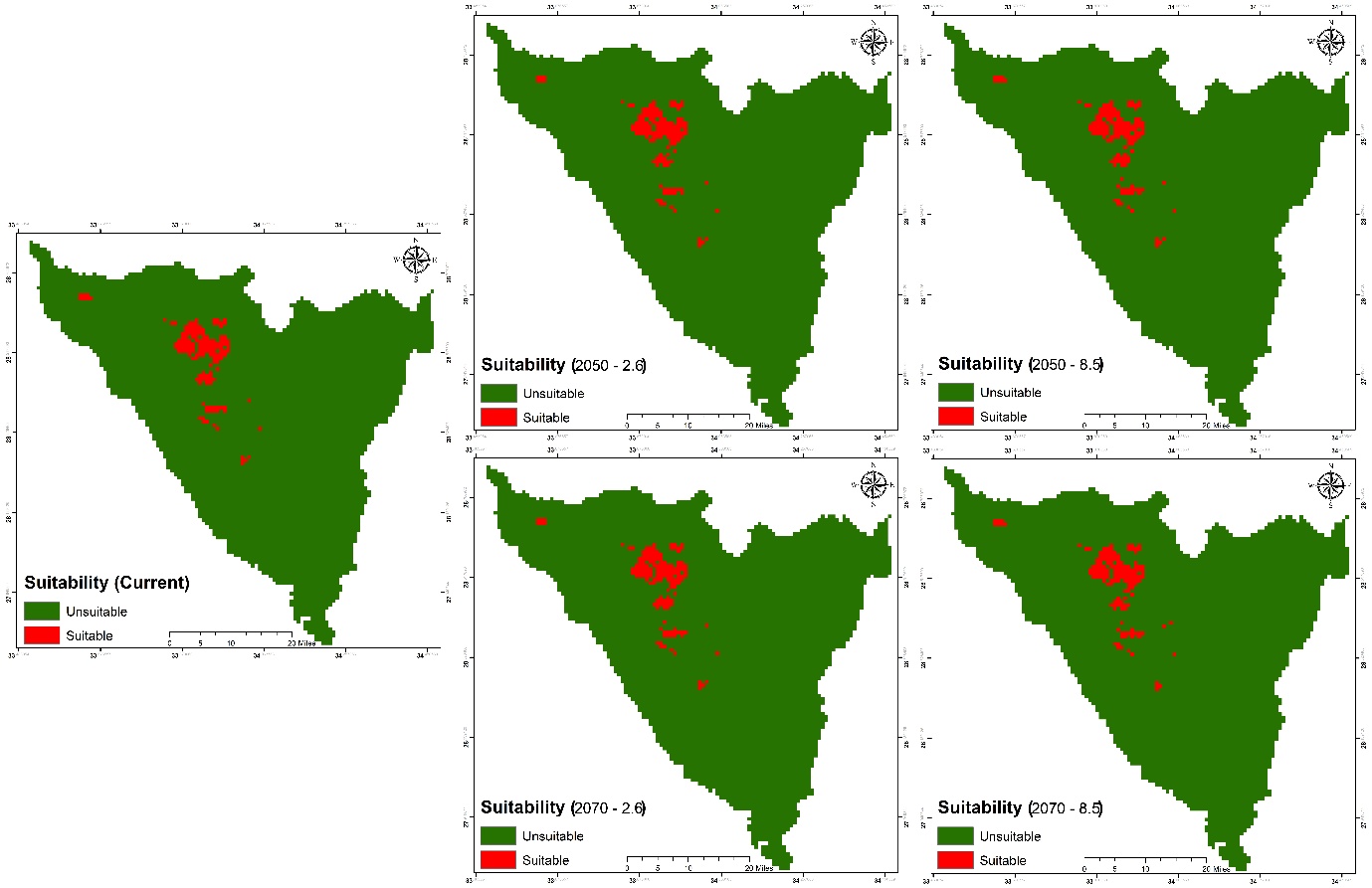
*

Fig. S14. Map of the current and future potential habitat for *Rosa arabica*


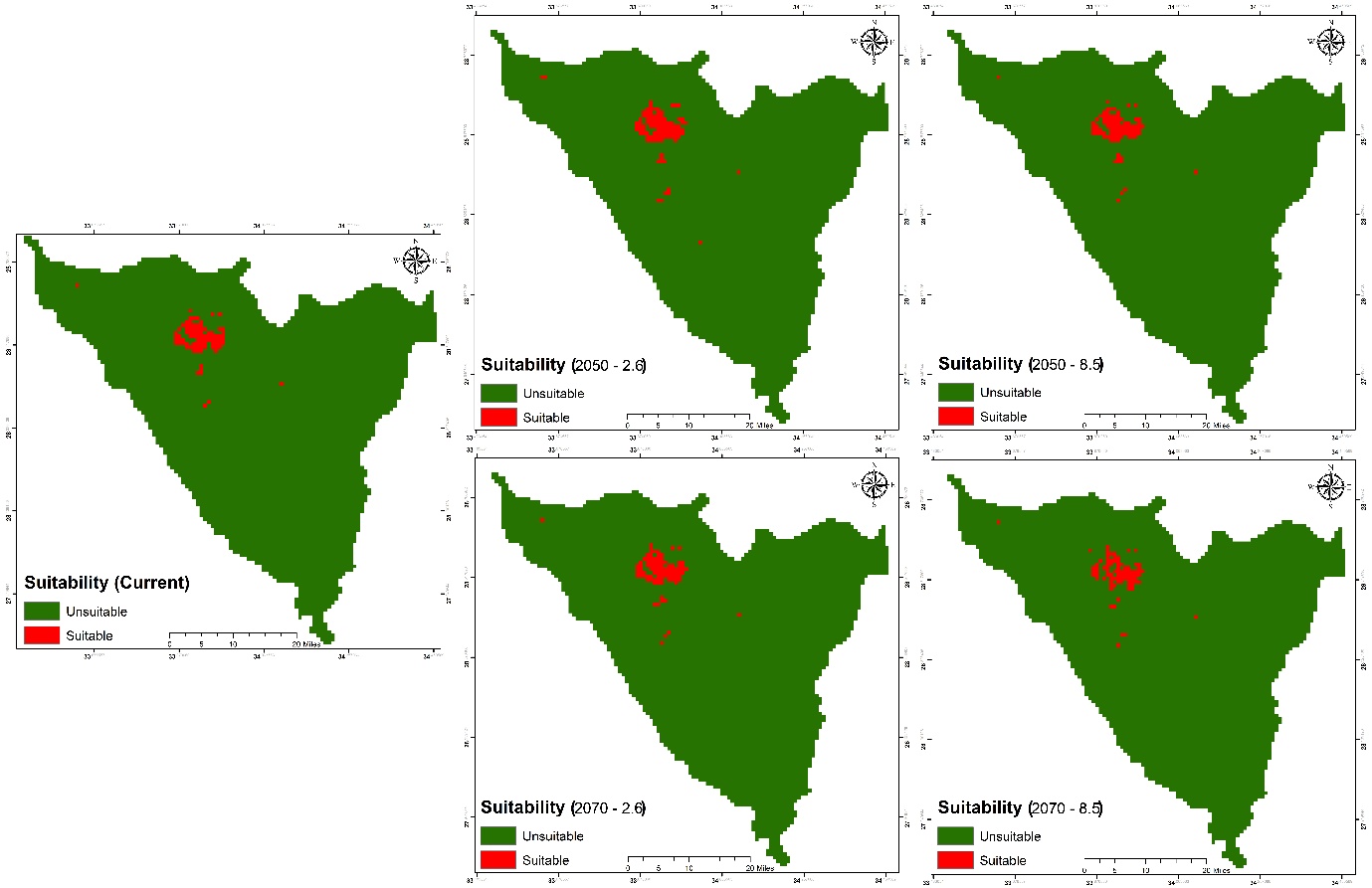


Fig. S15. Map of the current and future potential habitat for *Micromeria serbaliana*


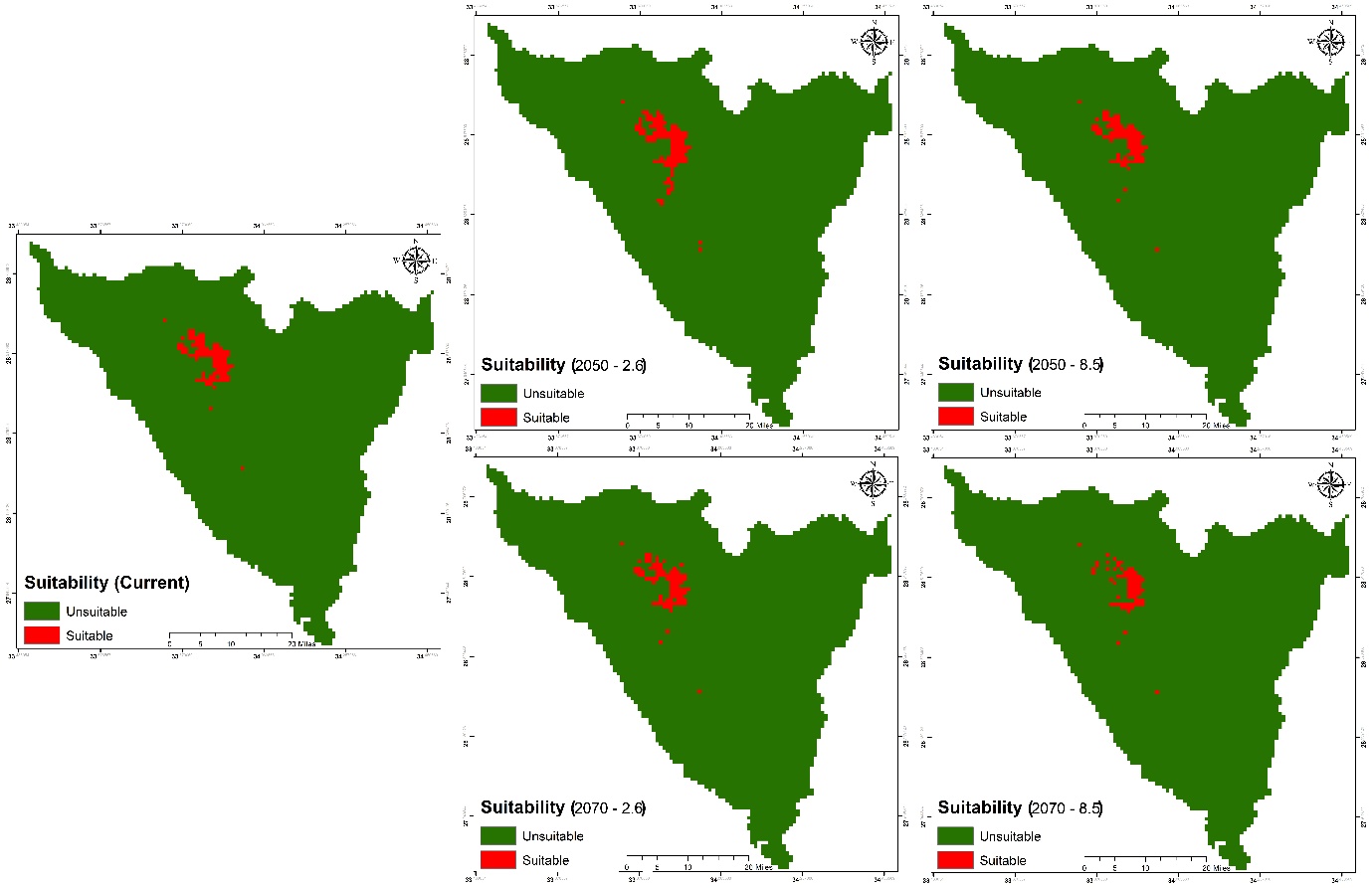


Fig. S16. Map of the current and future potential habitat for *Silene oreosinaica*
